# Autologous Hematopoietic Stem Cells Are a Preferred Source to Generate Dendritic Cells for Immunotherapy in Multiple Myeloma Patients

**DOI:** 10.3389/fimmu.2019.01079

**Published:** 2019-05-21

**Authors:** Prajakta Shinde, Sameer Melinkeri, Manas Kumar Santra, Vaijayanti Kale, Lalita Limaye

**Affiliations:** ^1^National Centre for Cell Science, Savitribai Phule Pune University Campus, Pune, India; ^2^Blood and Marrow Transplant Unit, Deenanath Mangeshkar Hospital, Pune, India

**Keywords:** multiple myeloma, stem cells, monocytes, dendritic cell vaccine, cytotoxic T cells

## Abstract

In multiple myeloma (MM), dendritic cells (DCs), and their precursors are prone to malignant cell-mediated regulation of function leading to low efficacy of DC vaccine. DCs taken directly from MM patient's body or derived from monocytes are fewer in numbers and are also dysfunctional. Here, we investigated the functionality of Hematopoietic stem cell-derived DCs (SC-DCs) from MM patients. Mature-MM-SC-DCs showed all essential functions like antigen uptake, allogenic T cells simulation and migration comparable to those derived from healthy donor (HD) samples. A comparison of Mo-DCs and SC-DCs obtained from the same MM patients' samples revealed that the expression of IL-6 was higher in the precursors of Mo-DCs leading to their impaired migration. In addition, expression of CCR7 which is responsible for DCs migration was found to be lower in MM-Mo-DCs. The chromatin permissiveness as observed by H3K4me^3^ histone modification at the Ccr7 promoter in MM-Mo-DCs was significantly lower than those in MM-SC-DCs. Levels of *Zbtb46*- a hall mark DC transcription factor mRNA was also found to be reduced in MM-Mo-DCs. Cytotoxic T cells generated from MM-SC-DCs from autologous naïve T cells exhibited reduced antitumor activity because the T cells were exhausted. Blocking of CTLA-4 on autologous T cells could partially restore T cell proliferation and activation. Thus, a combination of MM-SC-DC vaccine and anti-CTLA-4 antibody may serve as a better candidate for immunotherapy of MM. This study has implications in increasing the efficacy of cancer immunotherapy in MM.

## Introduction

Multiple myeloma (MM) is a hematological malignancy with the second most common propensity with mortality rate of 5.2 to 8 (1). MM is associated with the clonal expansion of malignant plasma cells that suppresses hematopoiesis, often leading to bone lesions, anemia, and renal complications (2, 3). Standard chemotherapy and radiation therapy, followed by autologous stem cell transplantation (AST), is the routine procedure administered to MM patients for treatment. Although this standard treatment may help patients to achieve remissions but eventually majority of MM patients show relapse of the disease, with incurable progression associated with immune deregulation (4, 5). Reversal of immune suppression and training the effector cells to specifically target malignant plasma cells would help in controlling the progression of the disease and improving the overall survival of patients (6).

Cancer immunotherapy using dendritic cells (DCs) has gained much attention as it has the potential to cure the disease, where other therapies have failed (7). DC vaccines are prepared by differentiation of precursor cells, i.e., monocytes, circulating in the patients' blood, or from differentiation of hematopoietic stem cells (8, 9). MM-associated tumor antigens are loaded onto the DCs so that they can activate a T cell response directed against cells expressing those antigens. The success of the DC vaccine depends on the immunogenicity of the final product (10, 11). The source population from which DCs are generated may differ from each other (12). Many studies, including ours, have suggested that DCs circulating in cancer patient's body and monocyte-derived dendritic cells (Mo-DCs) are functionally impaired (13–15). The precursors of these Mo-DCs secrete higher amounts of IL-6, leading to defective migration of Mo-DCs (16–18). In this study, we assessed the functional properties of DCs derived from a more primitive population, i.e., hematopoietic stem cells (HSCs) from MM patients. For this, we used a two step culture system (19). The first step involves expansion of the HSC pool toward DC precursors, and the second step involves differentiation of these precursors to mature DCs. Apheresis samples obtained from healthy donors (HD) or MM patients at remission were used for the generation of stem cell derived-DCs (SC-DCs).

We compared stem cell derived-DCs from MM patients (MM-SC-DCs) with those from healthy donors (HD-SC-DCs) for their morphology, phenotype and functions. These DCs were also utilized for autologous cytotoxic T cell (CTL) generation. These CTLs were characterized and tested for their targeted cell killing activity.

## Materials and Methods

### Ethical Approval

Written informed consents were obtained from healthy donors and multiple myeloma patients prior to collection of apheresis samples. All experimental procedures and informed consents were approved by the Institutional Ethics Committee (IEC) and Institutional Committee for Stem Cell Research (IC-SCR) of NCCS, and Deenanath Mangeshkar Hospital, in accordance with the Declaration of Helsinki. The study number from the ethics review board is NCCS/IC-SCR/2016-I/2.

### Sample Collection

Apheresis samples were collected from healthy donors or MM patients who were administered with GM-CSF for the mobilization of stem cells. Hematopoietic cells-enriched mononuclear cells were collected by using the COBE Spectra Apheresis System (Spectra Cell Separator; Terumo BCT Inc., Tokyo, Japan).

Upon completion of transplantation of the apheresis samples to the patients, 3 to 5 ml of samples leftover in the tubing were procured for this study from the transplantation unit.

- **Healthy donors**: Eight apheresis samples were collected from healthy donors who were donating cells for allogeneic stem cell transplantation.
- **Multiple myeloma patients:** Seven apheresis samples were collected from MM patients who were undergoing autologous stem cell transplant. The stage of the disease was diagnosed according to the International Staging System (ISS) for multiple myeloma. MM patients had undergone chemotherapy regimens to achieve remission of the disease. Samples were collected after 3–6 months of chemotherapy, when the patients were in remission.

### Dendritic Cell Generation

The details of two step method for generation of DC have been previously reported (19). Briefly, mononuclear cells (MNCs) from HD/MM apheresis samples were seeded for 1 h plastic adherence in tissue culture plates containing IMDM with 1% AB+ plasma in a CO_2_ incubator. After incubation, non-adherent cells were enriched for the stem cell population. These cells were expanded for 21 days using a combination of the growth factors, FMS-like tyrosine kinase 3-ligand (FLT3-L), 25 ng/ml; thrombopoietin (TPO), 10 ng/ml and stem cell factor (SCF), 20 ng/ml, to obtain DC precursor cells. Immature DCs were differentiated from the precursor population by using the granulocyte-macrophage colony-stimulating factor (GM-CSF, 50 ng/ml) + interleukin-4 (IL-4, 30 ng/ml) for 3 days, and GM-CSF (50 ng/ml) + Tumor necrosis factor-α (TNFα, 50 ng/ml) for 4 days. Maturation of DCs was induced by the addition of TNF-α, lipopolysaccharide (LPS), and CD40L at concentrations of 100 ng/ml each, for 48 h.

For generation of monocyte-DCs from MM apheresis samples, 10^7^ mononuclear cells were seeded/well in six-well plates. The cells were incubated for 1 h at 37°C in 5% CO_2_. The non-adherent and the loosely adherent cells were washed off with IMDM. The adherent cells were differentiated as described above. The MM-Mo-DCs and MM-SC-DCs were compared for the expression of IL-6 (Interleukin-6) and Zbtb46 (Zinc Finger And BTB Domain Containing 46), and for anti-trimethyl histone H3K4 modification at CCR7 promoter. All the cultures were maintained in IMDM supplemented with 5% AB^+^ plasma supplemented with penicillin (100 U/ml), streptomycin (100 mg/ml), and L-glutamine (2 mM) in a 5% CO_2_ incubator at 37°C.

### Maintenance of Cell Lines

HLA-A2-positive cell lines, U266B1 (Multiple myeloma), MDA-MB-231-luc-D3H2LN (breast cancer), HCT116 (colon carcinoma), A498 (renal cell carcinoma), and MCF-7 (breast cancer), were used in this study. K562, an HLA null cell line, was used as a non-specific control in *in vitro* CTL assays. All cell lines were maintained according to standard tissue culture practices.

### Flow Cytometry

Cells of interest were washed and blocked by PBS containing 1% BSA. For cell surface markers, antibodies against specific markers were added to cell suspensions, with their appropriate isotype controls being added in another tube. Antibody staining was done for 45 min on ice. For intracellular markers, fixation and permeabilization of cells was done prior to antibody staining, using the eBioscience Fix/Perm kit, as per the manufacturer's instructions. Stained cells were washed twice with ice-cold PBS and resuspended in 1% paraformaldehyde. Cells were acquired on FACS Canto II (BD, San Jose, CA, USA). Data were analyzed by FACS Diva (BD), and histogram overlays were prepared using FlowJo (LLC, Ashland Oregon).

### Functional Assays for DCs

- **Antigen uptake:** Antigen uptake by the process of endocytosis was assessed by using FITC-tagged Dextran (Molecular probe). Immature DCs were harvested and incubated with Dextran-FITC (20 μg/ml) either at 4°C (internalization control) or at 37°C (test), for 30 and 60 min. After completion of incubation, cells were extensively washed with ice-cold PBS containing 0.01% sodium azide and 1% BSA. Cells were then fixed with 1% paraformaldehyde (PFA) and acquired and analyzed on BD FACS Canto II.
- **Chemotaxis:** The migration property of mature DCs was assessed by an *in vitro* migration assay. 2 × 10^4^ cells in 100 μL of cell suspension were loaded on top of inserts (pores size 0.8 μm, BD Falcon). These inserts were placed in tissue culture wells containing IMDM either supplemented with or without rhCCL-19 (500 ng/mL). The culture was incubated for 3 h at 37°C with 5% CO_2_. After completion of incubation, migrated cells at the bottom of the well were harvested and counted using a hemocytometer and Trypan blue staining.
  To examine the role of autocrine IL-6 on migration of MM-Mo-DCs, anti-IL6 antibody at the concentration of 10 ug/ml was added from day 0 of the MM-Mo-DCs cultures, These MM-Mo-DCs were used for the chemotaxis assay.
  To check the specificity of interaction of chemokine receptors (CCR) with CCL-19, in migration assays SC-DCs were pretreated with anti-CCR1/anti-CCR3/anti-CCR5 or anti-CCR7 antibody (10 ug/ml) for 1 h.
- **Mixed lymphocyte reaction (MLR):** CD3^+^ T cells were sort-purified from peripheral blood-MNCs of healthy volunteers. T cells were labeled with CFSE dye (Molecular probe) as per the manufacturer's instructions. Co-cultures of mature DCs and labeled T cells in the ratio 1:10 (DC count = 10^4^ and T cell count = 10^5^) were carried out for 5 days. Labeled T cells without any added DCs were used as controls. CFSE dilution, used as an indication of proliferation of T cells, was assessed by flow cytometry.
- **ELISA:** IL12p70, IL10, and IFNγ in the culture supernatants was analyzed by using sandwich ELISA. Supernatants either from the *in vitro*-generated DCs or co-cultures of DCs and T cells were collected. The supernatants were then analyzed by ELISA for cytokine content, human-IL-10 and IFN-γ, using the BD Opt EIA ELISA kit (BD Biosciences, USA), and IL12p70 using the eBioscience ELISA kit, as per the manufacturers' instructions.

### Real Time PCR

Total RNA was extracted from 10^6^ cells using the Trizole reagent as per the manufacturers' instructions. The extracted RNA (1 μg) was reverse transcribed to cDNA using MMLV reverse transcriptase (Sigma Aldrich) and Random hexamers (Invitrogen), as per the instructions. qRT-PCR was performed with specific primers as listed below, using SyBr green with an AB Fast 7,500 platform.

**Table:** List of primers used for Real time PCR.

**No**.	**Gene name**	**Sequence (5′→** **3′)**
1	Human GAPDH forward	CGGATTTGGTCGTATTG
2	Human GAPDH reverse	GGAAGATGGTGATGGGA
3	Human CCR7 forward	GGTATGCCTGTGTCAAGATG
4	Human CCR7 reverse	GGTTGAGCAGGTAGGTATCG
5	Human IL6 forward	CAATGAGGAGACTTGCCTGG
6	Human IL6 reverse	TGGGTCAGGGGTGGTTATTG
7	Human Zbtb46 forward	AGAGTGCTGGTGATGCCTG
8	Human Zbtb46 reverse	ACAGGTCCGCATTTGAGTC

### Chromatin Immunoprecipitation (ChIP)

For the detection of H3K4me^3^ histone modification on the CCR7 promoter of mature DCs, 10^6^ cells were fixed in a 1% formaldehyde solution. Fixed cells were sonicated using a water-bath sonicator, and the assay was performed using the anti-trimethyl histone H3K4 antibody and ChIP assay kit (Milipore) as per the manufacturers' instructions. Quantitation of ChIP DNA (relative enrichment) was analyzed in triplicates by qRT-PCRs (ABI 7,500 fast). A list of the primers used for the CHIP assay is given below.

**Table:** List of primers used for ChIP-PCR.

**No**.	**Gene name**	**Sequence (5′→** **3′)**
1	Human GAPDH promoter forward	GCCAATCTCAGTCCCTTC
2	Human GAPDH promoter reverse	AAGAAGATGCGGCTGACTGT
3	Human CCR7 promoter forward	TGTATGTGGCAAAAGGG
4	Human CCR7 promoter reverse	CTCAGAAAACACCCAACA

### Generation of Anti-tumor CTLs:

Antigen pulsing of immature DCs was carried out by adding Keyhole Leukocyte antigen (KLH, adjuvant) and the target cancer cell lysate at concentrations of 50 and 100 μg/ml for 48 h, respectively. This tumor antigens-pulsed DCs were co-cultured with autologous sort-purified CD3+ CD8+ CD45RA+ naïve T cells. Sorting was performed on the BD Aria III platform; the gating strategy is given in Supplementary Figure S6. The co-cultures were maintained for 2 weeks with the addition of IL-2 (0.1 μg/ml) and IL-7 (5 μg/ml) every alternate day, and with the addition of fresh-pulsed DCs after a week, to provide re-stimulation to the CTLs. The target cancer cells used for CTL generation from HD samples in this study were- MCF7, MDA-MB-231-luc-D3H2LN, A498, HCT116 and U266B1 while U266B1 was used as the target cell line for CTL generation from MM samples.

### Functional Assays for CTLs

- **Assessment of activation by detection of the expression of surface markers:** Expression of CD 69 is the hallmark of T cell activation. Following 5–7 days of incubation, cells from CTL co-cultures were screened for the expression of CD69 using flow cytometry. Cells (2 × 10^5^ per tube) were stained as mentioned earlier. Dual positive cells expressing CD8 and CD69 were analyzed on FACS Canto II from BD.
- **Intra cytoplasmic staining for granzyme A, B and perforin:** *In vitro*-generated CTLs were tested for the expression of the serine proteases, granzyme A, B, and perforin within the intra cellular compartments. At the end of incubation, cells from the co-cultures were harvested and stimulated by PMA (40 ng/ml) and ionomycin (100 ng/ml), along with Golgi stop- Brefeldin A (1:1000 dilution), for 4 h. After incubation, cells were subjected to surface staining with CD8 for 30 min, followed by fixation and permeabilization. These cells were stained with antibodies against granzyme A, B and perforin for 60 min on ice. Stained cells were acquired and analyzed on FACS Canto II from BD.
- ***In vitro* CTL assay:** Target cells (10^6^/ml) were stained with 10 mM Calcein-AM for 30 min. These labeled target cells were then seeded with the effector cells (CTLs) in U-bottom 96-well microtiter plates, with T: E ratios ranging from 1:1 to 1:40, in triplicates. Target cells without the effector in complete medium (spontaneous release), and target cells in complete medium plus 2% Triton X-100 (maximum release) were used as controls. The HLA-null cell line, K562, was used as a negative control in these experiments. The *in vitro* killing activity was tested only on CTLs that were primed with the individual tumor cell line lysate. Unactivated T cell control could not be included, as the number of naïve T cell population that could be harvested from the available sample volume was limited (From 10^7^ MNCs we could get upto 5 × 10^5^ naïve T cells. The *in vitro* CTL assay requires more number of T cells as the ratio of target cells to effector cells gradually increases. Hence we could not use the unactivated naïve T cell control). After incubation at 37°C in 5% CO_2_ for 6 h, the supernatant was harvested and transferred into new plates. Fluorescence of the supernatant was measured using a microplate fluorimeter (excitation filter: 485–9 nm; band-pass filter: 530–9 nm) (20). Percent lysis was calculated by using the formula:

Percent lysis = [(test release − spontaneous release)/(maximum release – spontaneous release)] × 100.

### CTL Proliferation Monitored by Using Ki67

The Ki67 marker was used to monitor the proliferation of CTLs. Cells from DC-T cell co-cultures were harvested after 72 h of incubation, and stained with Ki-67-PE, and CD8-APC using antibodies. Ki67^+^ CD8^+^ dual positive cells were observed in the gated lymphocyte population.

### Autologous T Cells Profiling

Autologous CD3^+^ CD8^+^ T cells from both healthy donor and MM samples were profiled by staining with CD45RA and CD62L to identify T cell subsets (naïve, terminally differentiated effector cells, effector memory, and central memory T cells). In addition, cell surface staining of CTLA-4 was also performed on CD3^+^ CD8^+^ CD45RA^+^ T cells. The stained cells were acquired and analyzed by FACS.

### CTLA-4 Blocking

In some experiments of co-cultures of MM-SC-DCs and autologous naïve T cells, anti-CTLA-4 antibody, or IgG control antibody was added at the beginning. These co-cultures were maintained as described earlier with antibody concentration maintained at 10 μg/ml. After completion of 72 h of incubation, CTLs from the co-culture were assessed for proliferation and activation by monitoring Ki67 expression and CD69 expression by FACS.

### Statistical Analysis

Different experimental variables were compared and all the results were expressed as mean ± SEM. “N” represents the number of apheresis samples, i.e., biological replicates, and “n” represents the number of experimental replicates of a single apheresis sample. Statistical analysis was done and graphs were prepared using the Sigma stat software (Version 11). All statistical analyses of experiments between the two groups were evaluated by the one-way repeated measures ANOVA. *p*-values ≤ 0.05 were considered statistically significant [*p* ≤ 0.05(^*^), *p* ≤ 0.01(^**^), and *p* ≤ 0.001(^***^)].

## Results

### Patient Population

Age and gender of HD/MM patients and stage of the disease (for MM, at the time of first visit to clinic) are specified in Supplementary Figure S1A. In the present study, MM patients were given standard care chemotherapy followed by autologous stem cell transplant which gives faster hematopoietic recovery. Chemotherapy regimes given to individual patients are given in Supplementary Figure S1B. Apheresis samples from the MM patients were collected after 6 months of chemotherapy. These samples were used for dendritic cells generation.

### Cell Yield, Morphology, and Phenotype of SC-DCs From Healthy Donor and Multiple Myeloma Patients Were Similar

**Figure 1:**
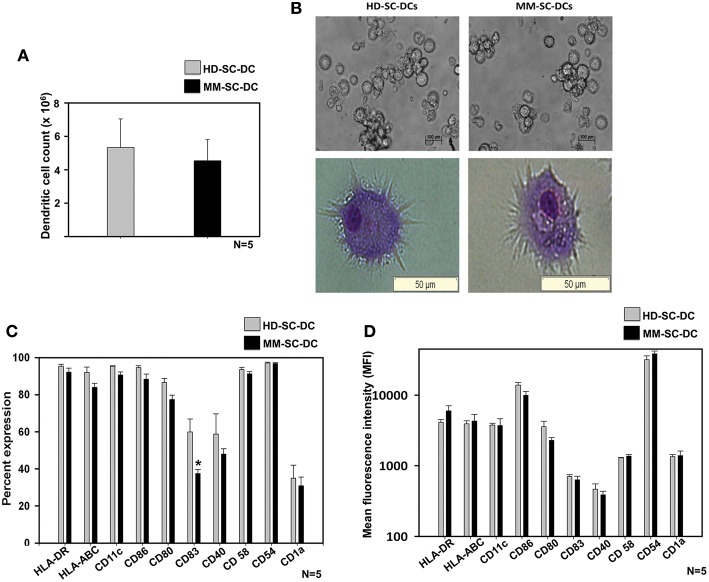
Yield, morphology, and phenotype of SC-DCs derived from HD and MM samples were similar. **(A)** The number of DCs obtained from HD and MM samples were equivalent. **(B)** Representative phase contrast images and Wright's-Giemsa stained cells of HD-SC-DCs (left panel) and MM-SC-DCs (right panel) are depicted. **(C)** The percent expression and **(D)** mean fluorescent intensities (MFI) for the DC-specific surface molecules were similar (except for percent of CD83). Data given are mean ± S.E.M *p* ≤ 0.5 (*).

A two-step method was used for the generation of DCs from stem cells of apheresis samples of HD and MM patients as described in material and methods. HSCs were expanded and terminally differentiated into DCs and were termed as HD-SC-DCs and MM-SC-DCs, respectively. They were systematically compared for morphology, phenotype, and functions. There was only a marginal difference in the absolute number of SC-DCs generated from 10^7^ MNCs from HD and MM apheresis samples (5.3 ± 1.7 × 10^6^ and 4.5 ± 1.2 × 10^6^, respectively, Figure 1A). Phase contrast imaging and Wright's-Giemsa staining of mature SC-DCs from both the sets exhibited characteristic morphology with long dendritic processes (Figure 1B). SC-DCs were assessed for the expression of various surface markers associated with mature DCs. As shown in Figure 1C, both the SC-DCs have more than 90% expression of HLA-DR, CD54, CD58, CD86, and CD11c. Expression of HLA-ABC and CD80 reached upto 80%, while the expression of CD40 was ~60% in both the SC-DCs, and the differences were not significant. Expression of CD83 was significantly lower in MM-SC-DCs as compared to HD-SC-DCs, but this difference was not reflected in their mean fluorescence intensity (MFI) values (Figure 1D). CD1a expression was around 20% in SC-DCs from HD and MM samples. MFI of all the DC-surface markers were also comparable between the HD and MM samples (Figure 1D). Representative histograms plots have been depicted in Supplementary Figure S2. Collectively, these results indicate that HD-SC-DCs and MM-SC-DCs exhibited typical mature DC morphology and cell-surface phenotype without any significant differences between them.

### Functionality of SC-DCs From HD and MM Source Was Equivalent

**Figure 2:**
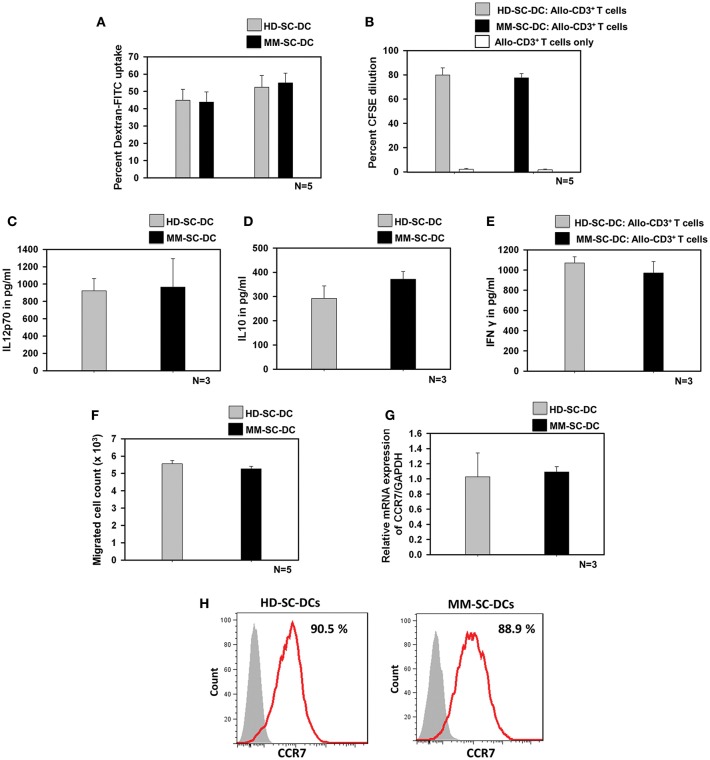
Functional characteristics of HD-SC-DCs and MM SC-DCs were comparable. **(A)** Percent dextran-FITC uptake by HD-SC-DCs and MM-SC-DCs were similar after 30 and 60 min. **(B)** Allogeneic T cells co-cultured with both SC-DCs showed similar proliferation, as observed by CFSE dye dilution in proliferating T cells. **(C)** Secretion of IL-12p70 and **(D)** IL-10 cytokines in culture supernatants of HD-SC-DCs and MM-SC-DCs showed marginal difference. **(E)** Concentration of IFNγ in supernatant of SC-DCs and allo-T cell co-cultures were equivalent, as assessed by sandwich ELISA. **(F)** Migration of SC-DCs toward CCL-19 from HD and MM samples was similar. Expression of CCR7 **(G)** mRNA and **(H)** protein was similar in SC-DCs of HD and MM samples, as observed by RT-PCR and FACS, respectively. Data given are mean ± S.E.M.

Antigen uptake by SC-DCs via receptor-mediated endocytosis was monitored by the uptake of dextran-FITC. HD-SC-DCs and MM-SC-DCs exhibited high and comparable dextran- FITC uptake following both, 30 and 60 min' incubation (Figure 2A). Representative histograms of dextran-FITC positive SC-DCs are given in Supplementary Figure S3. Mature and functional DCs induced the activation and proliferation of interacting T cells. This function was assessed by *in vitro* co-culturing of SC-DCs from HD or MM samples with allogeneic- CD3^+^T cells (allo-T cells) from the peripheral blood of unrelated healthy donors. The target T cells were labeled with the dye, CFSE. After 5 days of co-culture, T cells were analyzed for percent CFSE dilution. Figure 2B showed that T cells have equivalent cell proliferation rate in HD-SC-DC co-culture (79.8 ± 5.8%) as well as in MM-SC-DC co-culture (77.5 ± 3.5%). Representative FACS overlays are given in the Supplementary Figure S4. The data suggest that HD-SC-DCs and MM-SC-DCs have similar capacity to induce proliferation in T cells.

IL-12 and IL-10 are two important cytokines secreted by DCs that influence the immune response mounted by T cells. We analyzed the secretion of these two cytokines in SC-DC culture supernatants. It was found that MM-SC-DCs and HD-SC-DCs had secreted similar amounts of IL-12p70 (Figure 2C) and IL10 (Figure 2D) in their culture supernatant. For assessing the levels of IFNγ, the supernatants of SC-DC, and CD3^+^ T cell co-cultures were collected and analyzed by ELISA. The levels of IFNγ in the supernatants of CD3^+^ T cell co-cultures either with MS-SC-DC or HD-SC-DC were also comparable (Figure 2E). Next we examined the migration function of SC-DCs from HD and MM samples. Migration of DCs toward a chemokine gradient is an essential functional property for initiation of antigen specific immune response. DCs migration to local lymph node is governed by the chemokine receptor CCR7 and CCL-19 interaction. As seen in Figure 2F, MM-SC-DCs showed similar migration toward CCL-19, as compared to HD-SC-DCs. To further confirm this observation, we analyzed CCR7 marker expression on MM-SC-DCs and HD-SC-DCs.

CCR7 expression in SC-DCs of HD and MM samples was equivalent at the transcript as well as protein levels (Figures 2G,H). Specificity of CCR7 toward CCL19 was tested by blocking of CCR receptors like CCR1, CCR3, CCR5, and CCR7 while performing *in vitro* migration assay. The migration of SC-DCs was abrogated on blocking of CCR7 only (Supplementary Figure S5) highlighting the fact that the DC migration is dependent on CCR7 and CCL-19 interaction. Taken together, these data show that, SC-DCs derived from MM-HSCs have all the functional characteristics similar to those shown by SC-DCs derived from normal healthy HSCs, thus indicating functional equivalence.

### MM-Mo-DCs Showed Impaired Migration Than MM-SC-DCs Derived From the Same MM Samples

In many types of cancers including multiple myeloma, autocrine secretion of IL-6 is known to inhibit DCs migration function (14–17). Our earlier studies had revealed that, DCs generated from monocytes of MM samples were impaired in migration function and CCR7 expression (14). In contrast, here we found that these functions were not compromised when DCs were generated from stem cells (MM-SC-DCs), with these showing migration (Figure 2F) and associated *Ccr7* expression, equivalent to HD-SC-DCs (Figures 2G,H). To gain a deeper insight into this discrepancy, between functions of MM-Mo-DCs and MM-SC-DCs, both types of DCs were generated from the same MM samples' and were then compared.

**Figure 3:**
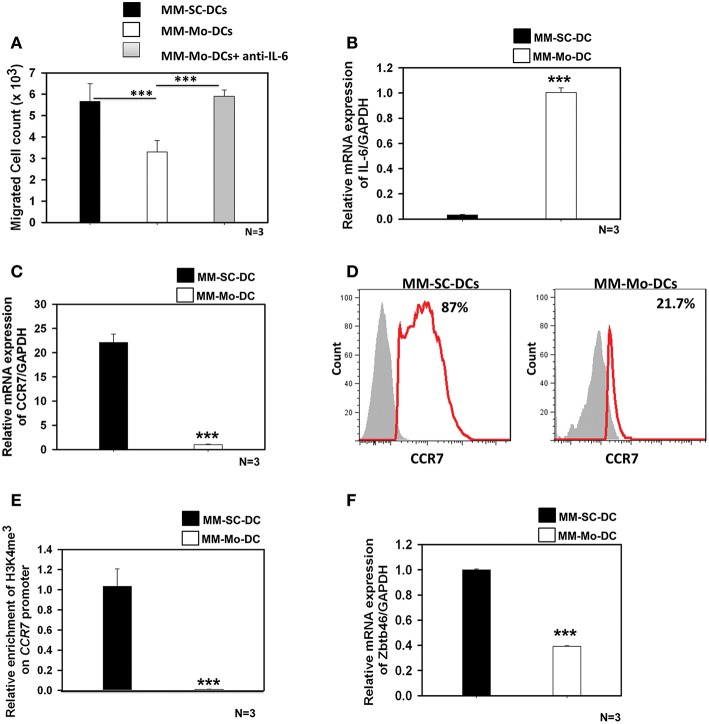
MM-Mo-DCs and MM-SC-DCs generated from the same MM samples showed differences at the molecular level. **(A)** MM-Mo-DCs showed significantly reduced migration toward CCL-19, as opposed to MM-SC-DCs. When MM-Mo-DCs were differentiated in presence of anti-IL6 antibody, their migration capacity was restored. **(B)** Expression of the IL-6 mRNA in precursors of MM-SC-DCs was significantly lower, as compared to that of MM-Mo-DC precursors. Expression of CCR7 at **(C)** the transcript and **(D)** the protein level was significantly lower in MM-Mo-DCs in comparison with MM-SC-DCs as analyzed by Real time PCR and FACS, respectively. **(E)** Relative enrichment of H3K4me^3^ at the Ccr7 promoter in MM-SC-DCs was significantly higher, as compared to MM-Mo-DCs. **(F)** Levels of the mRNA of transcription factor Zbtb46 were significantly higher in MM-SC-DCs, as opposed to those in MM-Mo-DCs. Data given are mean ± S.E.M *p* ≤ 0.001 (***).

As seen in Figure 3A, migration of MM-Mo-DCs was significantly reduced as compared to those of MM-SC-DCs, from same MM samples. When precursors of MM-DCs were analyzed for autocrine expression of IL-6 at transcript level, it was found to be negligible in SC-DCs, as compared to Mo-DCs (Figure 3B). The migration capacity of MM-Mo-DCs was restored when they were differentiated in presence of anti-IL-6 antibody to block the effect of autocrine IL-6 during their differentiation (Figure 3A). This observation suggested that higher expression of autocrine- IL6 might be one of the factors contributing to the observed reduced migration of MM-Mo-DCs as compared to MM-SC-DCs.

Epigenetic modification, in the form of DNA methylation and/or posttranslational modifications of histones regulates gene expression at the transcription level by governing the chromatin accessibility. The influence of epigenetic modification on DC functionality is poorly understood. There are reports suggesting that the migration of DCs is influenced by histone modification at the *CCR7* locus (21, 22). In contrast to MM-SC-DCs, expression of CCR7 at both transcript (Figure 3C) and protein levels (Figure 3D) was significantly lower in MM-Mo-DCs. We examined the abundance of H3K4me^3^ at the *CCR7* promoter of Mo-DCs and SC-DCs by ChIP-PCR. The chromatin was isolated from mature SC-DCs and Mo-DCs from same MM samples and ChIP was done using anti-H3K4me^3^. Real time-PCR revealed that recruitment of H3K4me^3^ was increased 10-fold at the promoter of the *CCR7* gene in MM-SC-DCs, as compared to MM-Mo-DCs (Figure 3E). These data indicated that reduced migration of Mo-SC-DCs might be due to lower CCR7 expression, governed by lower accessibility of this gene for transcription.

The Zbtb46 transcription factor is selectively expressed in the classical dendritic cell (cDCs) lineage and its progenitors. It is required for the development of cDCs (23). This transcription factor is down regulated in DCs and their progenitors when they are exposed to tumor microenvironment, i.e., factors secreted by a tumor (24). When we analyzed the expression level of Zbtb46 in MM-SC-DCs and MM-Mo-DCs obtained from the same MM sample, MM-Mo-DCs showed significantly reduced levels of mRNA for this transcription factor (Figure 3F). Thus, prior continuous exposure of precursors of Mo-DCs to a tumor microenvironment may have altered their immunocompetence, leading to their dysfunction.

### HD-SC-DCs Could Generate Antitumor Cytotoxic T Lymphocytes

A potent DC vaccine should generate the effector T cells having tumor killing activity, i.e., CTLs. In order to confirm whether SC-DCs from apheresis samples could generate potent autologous CTLs similar to those generated from Cord blood derived SC-DCs (25), initial experiments were done using HD-SC-DCs. We used an HLA-A2 restricted system for the CTL assays. Apheresis samples from healthy donors were screened for expression of the HLA-A2 molecule, and used for CTL generation. Co-cultures of DCs and naïve T cells were set up. Briefly, DCs were generated from these samples and pulsed with individual HLA-A2^+^ target cancer cell lines, MDA-MB-231-LUC-D3H2LN, MCF7, A498, or HCT116. Antigen- pulsed HD-SC-DCs were co-cultured with autologous naïve CD8^+^ T cells. The activation of T lymphocytes induces the expression of cell surface marker CD69. This marker was detected on the cell surface of CTLs using flow cytometry. It was clearly evident that CTLs generated from HD-SC-DCs showed expression of CD69 (Supplementary Figure S7A). Intra cytoplasmic staining was performed to detect the presence of proteases granules. We found that CTLs generated form HD-SC-DCs produced granzyme A, granzyme B, and perforin (Supplementary Figure S7A). The presence of serine proteases confirms the killing potential of CTLs against their target cells. The expression of these molecules ranged from 69 to 95%. These CTLs also secreted substantial amounts of IFN-γ in the co-culture supernatant (Supplementary Figure S7B). The CTLs generated from HD-SC-DCs were able to kill breast cancer triple negative MDA-MB-231-LUC-D3H2LN cells (Supplementary Figure S8A), luminal A breast cancer MCF7 cells (Supplementary Figure S8B), renal cancer A498 cells (Supplementary Figure S8C) and colon cancer HCT-116 cells (Supplementary Figure S8D). The specificity of target cell lysis by the CTLs was evident from the fact that, the percent target cell lysis was increased as the target: effector ratio increased from 1:1 to 1:40. Further, negligible cell killing of a non-specific target, i.e., K562 cells (HLA-null) was observed confirming the HLA-restricted killing activity of the CTLs.

### MM-SC-DCs Could Generate Antigen-Specific CTLs, but Were Compromised as Compared to CTLs From HD-SC-DCs

**Figure 4:**
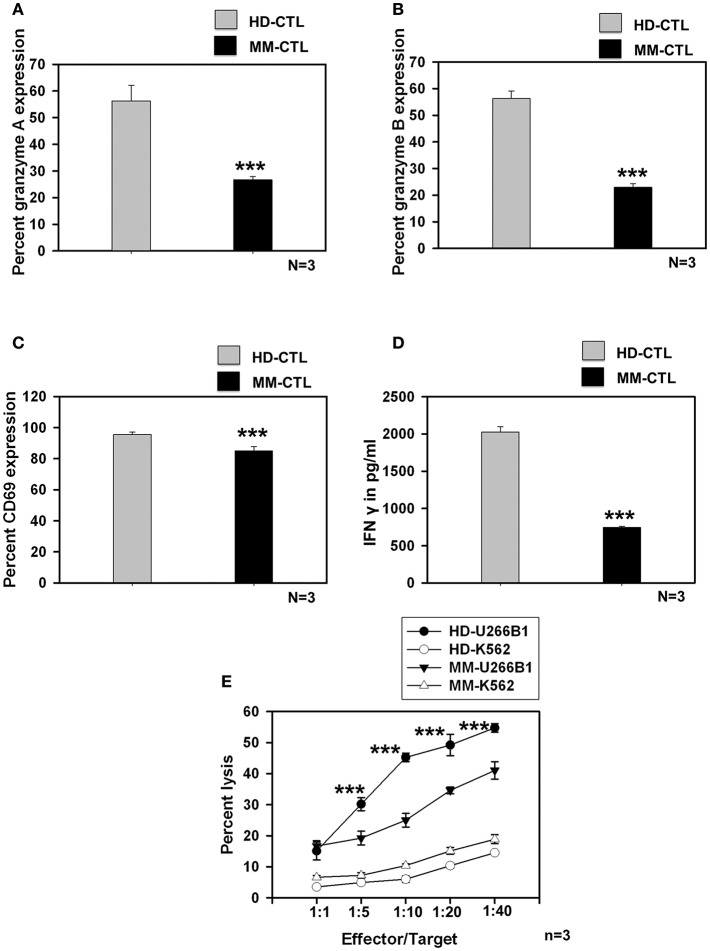
CTLs generated from MM-SC-DCs were compromised, as compared to those from HD-SC-DCs. The percent expression of **(A)** granzyme A **(B)** granzyme B, and **(C)** CD69 was significantly reduced in the MM CTLs, as against that in the HD CTLs. **(D)** The concentration of IFNγ in the SC-DC- CTL co-cultures of MM samples was significantly lower, as compared to those of HD samples. **(E)** The target cell killing activity of MM-CTLs tested against the U266B1 cell line was significantly reduced as compared to HD-CTLs. Killing of non-specific target cell line K562 was negligible in both HD and MM CTLs. Data given are mean ± S.E.M *p* ≤ 0.001 (***).

We further studied whether SC-DCs from MM samples could also generate potent CTLs, using the lysate of the HLA-A2^+^ multiple myeloma cell line, U226B1. CTL generation, characterization and assays for testing their function were performed on HLA-A2 positive HD and MM samples as described earlier. Contrary to our expectations, MM-CTLs generated from MM-SC-DCs were functionally defective and showed signs of exhaustion, as compared to HD-CTLs generated from HD-SC-DCs. The expression of granzyme A (Figure 4A), granzyme B (Figure 4B), the surface activation marker CD69 (Figure 4C), and secretion of IFN-γ (Figure 4D) in the culture supernatant were found to be significantly declined in the CTLs from MM samples as compared to the HD samples. Representative dot plot for the expression of CTLs markers are given in Supplementary Figure S9. Further, *in vitro* CTL activity against the target U266B1 cell line was significantly reduced in MM samples, as compared to HD-CTLs. Killing of K562 (a non-specific cell line) was negligible, and was not significantly different between HD and MM-CTLs (Figure 4E).

### Naïve T Cells in MM Showed Exhaustion, Which Could be Rescued Using a CTLA-4 Blocking Antibody

**Figure 5:**
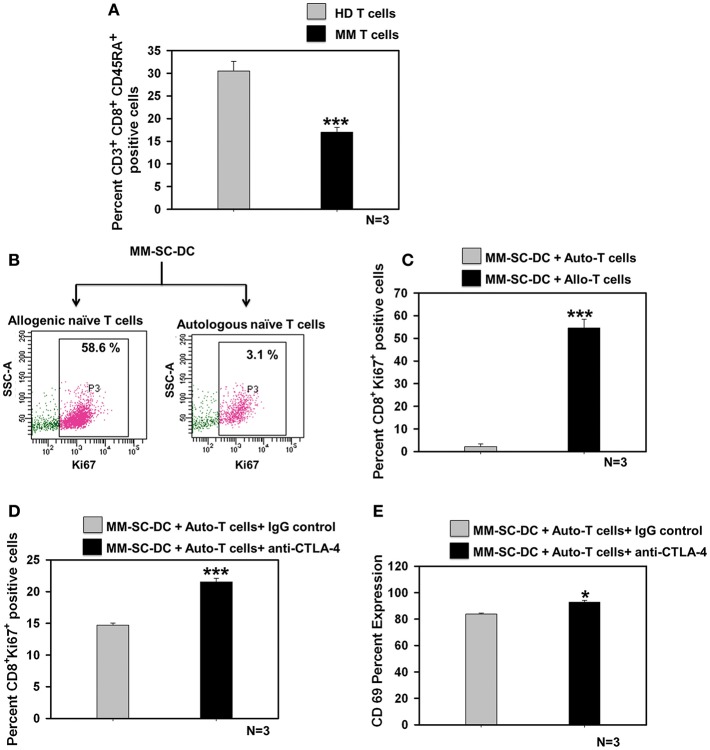
Exhaustion of naïve T cells of MM could be rescued by blocking the CTLA-4 molecule. **(A)** Percent of naïve T cells in the MM samples was significantly lower as compared to HD samples. **(B)** Representative FACS profile of Ki-67 positive cells and **(C)** Data from three different samples of MM-SC DCs and allo/auto- T cell co-cultures is depicted. Percent Ki-67 positive cells were significantly lower in MM-SC-DCs co-cultured with autologous T cells as compared to co-cultures with allogeneic T cells. **(D)** Blocking of CTLA-4 molecule in the co-cultures of MM-SC-DCs and autologous T cells enhanced Ki-67 positive population as compared to co-cultures where IgG isotype antibody was added. **(E)** T cells activation in co-cultures of MM-SC-DCs and autologous naïve T cells was significantly increased after blocking of CTLA-4 receptor. Data given are mean ± S.E.M, *p* ≤ 0.05 (*) *p* ≤ 0.001 (***).

The percent of CD8^+^ naïve T cell population in the MM samples was significantly lower as compared to that in healthy donor samples (Figure 5A). T cell population in apheresis samples were further examined for effector and memory phenotype using CD45RA and CD62L markers. No difference was seen for terminally differentiated effector cells, effector memory, and central memory cells (Supplementary Figure S10). To examine whether functional impairment of MM-CTLs was due to a defect in MM-SC-DCs or autologous T cells obtained from MM samples. Co-cultures of MM-SC-DCs with autologous naïve T cells or allogeneic naïve T cells obtained from HLA-A2^+^ healthy donor samples were studied. Expression of Ki-67 on T cells was monitored as an indication of initiation of T cell proliferation and activation. It was observed that T cells from an allogeneic source have higher level expression of Ki-67, as compared to T cells from an autologous source (Figures 5B,C). The above data clearly indicated that, even though SC-DCs obtained from MM samples were functional and immunocompetent, MM-SC-DC vaccine may not be superior for tumor regression in MM patients, since autologous T cells are exhausted. Exhausted T cells in chronic infections and cancer show expression of the surface molecule, CTLA-4 (cytotoxic T-lymphocyte-associated protein 4, Supplementary Figures S11A,B). Binding of CTLA-4 with its ligand initiates a signaling cascade that lowers the effector function of T cells. To test if blocking of CTLA-4 would result in increased proliferation and activation of autologous T cells' we performed MM-SC-DC and autologous T cell co-culture in the presence or absence of anti-CTLA-4 antibody. Expression of Ki-67 and CD69 markers on CTLs was significantly increased in the presence of the CTLA-4 blocking antibody during the co-cultures (Figures 5D,E). However, even after blocking of CTLA-4, the percent of Ki-67 positive cells reached only upto 21% which is still lower as compared to Ki-67 positive cells in healthy allo-naïve T cells and MM-SC-DCs co-cultures (upto 58%, Figure 5C). Thus, indicating that blocking of CTLA-4 only partially restores the autologous T cell proliferation and activation. In other words, SC-DCs vaccines treatment of MM, may be effective if when used in combination with anti-CTLA-4 antibody.

Taken together our data show that DCs generated from stem cells are more potent than the DCs generated from monocytes of MM patients. Stem cell derived-DCs from apheresis samples from healthy donor could generate functional CTLs. Though the SC-DCs from MM samples were functional, autologous T cells showed signs of exhaustion, which could be partially rescued with the help of the immune check-point inhibitor CTLA-4. Hence, for treatment of MM, the potency of stem cell-derived DC vaccine could be enhanced when they are used in a combination with the checkpoint inhibitor, CTLA-4.

## Discussion

In multiple myeloma, activation of an anti-tumor immune response completely eliminates the disease while compromised immunity contributes to its aggression (26). Malignant cells adversely affect immune system leading to its deregulation. Training or modulating the immune system to combat malignant cells with the help of a DC vaccine could be one of the effective solutions to this problem. Therefore, immunogenic DC vaccine preparation is a crucial part of the DC based cancer immunotherapy (27). There are reports of DC vaccines being used for the treatment of MM. However, the efficacy of these vaccines is still below the expectation (28). In order to increase the efficiency some investigators have attempted a combination of DC vaccine with the drug Lenalidomide for treatment of MM (29) and colon cancer (30) in murine model. In yet another study pomalidomide and dexamethasone were combined with DC vaccine and tested in murine MM model (31). Vo et al. (32) have also shown that addition of Lenalidomide in different concentrations *in vitro* on Mo-DCs obtained from multiple myeloma patients improved the functionality of DC.

In our previous study, we had shown that some of the functions of monocytes-derived DCs from MM patients' samples were compromised (15). To evaluate if DCs derived from stem cells of MM patients' samples are similarly impaired, the present study was undertaken. HSCs from apheresis samples were expanded into DC precursors and differentiated into mature DCs using a two step method developed in our lab. It was observed that HD-SC-DCs and MM-SC-DCs were equivalent with respect to morphology, phenotype, and functions. In the phenotype the percent expression of CD83 however was significantly lower in MM-SC-DCs as compared to HD-SC-DCs. CD83 is an important costimulatory molecule expressed on DCs (33). Though the percent values of CD83 were low, the MFI values for CD83 of MM-SC-DCs were comparable with that of HD-SC-DCs (Figure 1D). This difference was not seen at transcript level also (Supplementary Figure S12). Nonetheless it is worth looking into the mechanism behind low expression of CD83 in MM-SC-DC at protein level.

Cord blood (CB) being an easily available source of HSCs, CB-HSCs derived-DCs are well-studied. The anti-tumor activity of stem cell-derived DCs from cord blood or mobilized peripheral blood were tested in Phase I and II clinical trials with promising outcomes of generation of tumor-specific immunity against metastatic melanoma (9, 34, 35). Titzer et al. (36) reported that stem cell derived-DCs can induce clinically-relevant humoral and cellular idiotype-specific immune responses in advanced MM patients. Currently, a two-arm phase I randomized trial is underway (NCT01995708) to test the efficacy of autologous stem cell-derived DCs transfected with mRNA encoding TAAs, for the treatment of multiple myeloma patients. These studies strongly suggest that, stem cells could be used to generate large numbers of functional DCs for cancer immunotherapy.

To gain deeper insights into the observed differences in the functionality of Mo-DCs and SC-DCs from MM patients, a paired sample study was performed, which revealed that the precursor of MM-Mo-DCs had higher autocrine IL-6 secretions as compared to MM-SC-DCs. It is well-documented that high expression levels of IL-6 in DCs are responsible for defects in migration in other types of cancers including, cervical cancer (17), breast cancer (18), and ovarian cancer (37). Blocking of IL-6 during differentiation of MM-Mo-DCs resulted in the gain of migration function. Migration in MM-Mo-DCs was significantly lower when all four sets were compared (Supplementary Figure S13). DCs that can initiate an antitumor immune response are defined by chemokine receptor CCR7, the transcriptional factor Zbtb46, and the Flt3L and Kit receptors (38). In MM-Mo-DCs, expression of CCR7 was reduced as compared to MM-SC-DCs. Lower abundance of H3K4me^3^ at the CCR7 promoter might also be responsible for lower CCR7 expression and its associated migration in MM-Mo-DCs as compared to MM-SC-DCs. The signature DC transcription factor, Zbtb46, was also lower in the MM-Mo-DCs, as opposed to MM-SC-DCs, suggesting that the mature DC population may vary in its intrinsic properties if these cells are derived from two distinct precursor populations from the same patient. Thus, our findings suggest that SC-DCs are potential candidates for use as cancer vaccines in MM. Though here our study is limited to migration function of SC-DCs, still further molecular characterization of other functions like antigen processing and presentation remains to be looked into.

Previous studies have reported that tumor cells and their microenvironment convert myeloid cells into immunosuppressive cells (39, 40). Peripheral blood monocytes which are taken directly from MM patients' body have been exposed to a tumor regulatory microenvironment. Mo-DCs obtained from these monocytes were therefore found to be impaired. For generation of SC-DCs, HSCs were cultured in the presence of FLT3-L, SCF and TPO, which are known to promote the expansion of DC-precursors (41). These precursor cells were then differentiated into mature and functional DCs as seen by up regulation of costimulatory molecules in all four groups (Supplementary Figure S14). In cancer patients, presence of tumor cells or tumor-derived cytokines, or other factors, leads to defective differentiation, and functions of DCs (42). However, this was not the case for *ex vivo*–generated SC-DCs, since a tumor-associated microenvironment was completely absent during expansion and differentiation of the DCs precursors. Exposure to prolonged culture conditions and absence of tumor microenvironment during *in vitro* differentiation may have contributed to similarities observed in functional characteristics of MM-SC-DCs to HD-SC-DCs. Reports also suggest that DCs obtained from *in-vitro* differentiation of stem cells are more immunocompetent than the peripheral blood circulating DCs or monocyte-derived DCs of cancer patients (43, 44). We were also able to obtain functional DCs from stem cells, but not from monocytes of same MM samples. The ability of SC-DCs from MM patients for their anti-tumor response under *in vivo* situation is worth studying using humanized mouse model of multiple myeloma.

Generally for studying antitumor CTL generation from DC vaccine, a HLA-A2 restricted system is employed by various investigators (45–47). Similarly here we have focused on CD8^+^ T cells as we are using HLA-A2 restricted system. We have screened both healthy and MM samples for HLA-A2 positivity and used U266B1 cell line which is not only HLA-A2 positive but also a model cell line for MM. Peptide antigen generated from MUC1 antigens are known to be cross presented by DCs in the form of a complex with HLA-A2^+^ (48). MUC 1 is highly expressed in U266B1 (49). Cytotoxicity assay in our study provides evidence that the killing of target cancer cell lines is HLA dependent. However, alloreactivity and tumor antigens dependent killing by the CTLs primed by tumor antigen pulsed SC-DCs remains to be verified.

CD4^+^ T cells also have a major role to play in CD8^+^ T cells stimulation. It is worth looking into how MM-SC-DCs contribute to anti- tumor CD4 generation from naïve CD3 cells. These studies form a part of our future work plan.

Our data indicated that CTLs primed from HD-SC-DCs of apheresis samples from healthy donors exhibited efficient killing of cancer cell lines *in vitro*. However, CTLs from MM-SC-DCs exhibited reduced killing activity due to exhaustion of autologous T cells. Recently, Leone et al. (50) have shown that BM of MM patients, have a CD8^+^ T cell population that expresses Foxp3, produces IL-10 and TGF-β, and exerts pro-tumor activity. Antigen specific T cell activation requires T cell receptor (TCR) engagement with the antigen/MHC complex followed by costimulatory signal. After antigen recognition, co-stimulatory molecules on APCs interact with their binding partner on T cells and initiate T cell proliferation and activation. Prolonged antigen exposure in chronic diseases is responsible for T cell exhaustion. Such T cells express the surface marker, CTLA-4, which arrests T cell activation. Cytotoxic T lymphocyte–associated antigen 4 (CTLA-4) and CD28 are the two receptors on T cell. Both of these receptors bind to co-stimulatory molecules B7.1 and B7.2 on APCs. CD28 acts as an activator of T cell proliferation by binding to B7 and promoting IL-2 production and thus initiating T cell activation. Whereas, CTLA-4 act as the negative regulator, it inhibits T cell activation by inhibiting IL-2 production and cell cycle progression of T cells (51). CTLA-4 signal is crucial for Foxp3+ Treg development (52, 53). There are reports that Treg cells promote tumor progression. T_reg_ cells hamper immune surveillance against cancer and prevent the development of effective antitumor immunity in tumor-bearing patients, and promote tumor progression. Therefore, for successful cancer immunotherapy, it is required that T regulatory cells should be kept suppressed in the tumor microenvironment (54).

We studied the effect of blocking of CTLA-4 on T cell proliferation and it was observed that it partially restored the proliferation in T cells and their activation. However, the T cell phenotype analysis that we have performed in the study is limited to CD45RA and CD62L. More detailed study that defines MM associated memory T cell exhaustion using CD45RA and CCR7 needs to be undertaken. In addition it is also important to further characterize the T cells by staining with key markers such as PD-1, TIM-3, TIGIT, and LAG-3 for demonstration of T cell exhaustion.

Blocking of CTLA-4 using antibodies has been reported to reactivate effector function of T cells from their exhausted state (55, 56). Previously, metastatic melanoma and ovarian carcinoma patients were vaccinated with DC vaccine or GM-CSF secreting tumor cells (57). In these patients, the T cell- mediated anti-tumor response against melanoma, and ovarian carcinoma was increased after the administration of an anti-CTLA-4 antibody. In other words, their data showed that, the efficiency of CTLs generated by a cellular vaccine was increased by administrating the cancer patients with anti-CTLA-4 antibody. In a preclinical study using mouse model, it was shown that check point inhibitors like CTLA-4 and PD-1 activate CD103^+^ DCs and increase IL-12 secretion by them, leading to an increase in the anti-tumor immune response (58). Vo et al. (59) have used a dual combination of lenalidomide and programmed death (PD)-1 blockade to enhance efficacy of DC vaccine in MM in murine model.

A combination of a DC vaccine with a CTLA-4 blocking antibody (ipilimumab) was studied in a Phase II clinical trial by Wilgenhof et al. (60). In the cohort, 51% of the patients achieved complete or partial remission of melanoma and the overall response rate was 38%. Currently, numerous clinical studies on the effect of the combination of DC vaccines with immune check point inhibitors on cancer patients are underway (www.clinicaltrials.gov). We also observed in present study that activation of T cells and their proliferation was partially regained by blocking the CTLA-4 receptor in MM-SC-DCs and autologous T cells co-cultures. In conclusion, our findings demonstrated that combining MM-SC-DCs with a check point inhibitor could be a preferred DC-vaccine strategy for MM cancer immunotherapy.

Many clinical trials with DC-based cancer treatment modalities have shown that their clinical efficacy still needs improvement. Our findings would be helpful in enhancing the efficacy and feasibility of personalized cancer immunotherapy using DC vaccines for multiple myeloma.

## Ethics Statement

Written informed consents were obtained from healthy donors and multiple myeloma patients prior to collection of apheresis samples. All experimental procedures and informed consents were approved by the Institutional Ethics Committee (IEC) and Institutional Committee for Stem Cell Research (IC-SCR) of NCCS, and Deenanath Mangeshkar Hospital, in accordance with the Declaration of Helsinki.

## Author Contributions

PS carried out the experiments and analyzed the data. PS and LL were involved in the interpretation of the data. MS contributed to epigenetic study design. SM provided clinical samples. PS, LL, and VK wrote the manuscript. LL conceived and designed the study. All authors reviewed the data, and approved submission of the manuscript.

### Conflict of Interest Statement

The authors declare that the research was conducted in the absence of any commercial or financial relationships that could be construed as a potential conflict of interest.

## Supplementary Material

Supplementary Figure S1 Healthy donors and multiple myeloma patients' characteristics. **(A)** Details of age and sex of the healthy donors, and stage of the disease at the time of the first visit (for MM patients) is depicted for 8 HD and 7 MM samples. **(B)** chemotherapy regimens of 7 MM samples is tabulated.

Supplementary Figure S2 Cell surface marker expression on HD-SC-DCs and MM-SC-DCs was comparable. Representatives histogram of DC lineage surface marker (HLA-DR, HLA-ABC, and CD58 in green color; CD86, CD80, CD83, and CD54 in orange color; CD11c, CD40, and CD1a in red color lines) along with their appropriate isotype controls (Gray color filled) are depicted.

Supplementary Figure S3 MM-SC-DCs and HD-SC-DCs show equivalent dextran-FITC uptake. A representative FACS histogram overlay of dextran- FITC uptake profile from HD-SC-DCs and MM-SC-DCs is depicted. Green line shows uptake at 37°C while the blue line shows uptake at 4°C. Gray shaded histogram shows unstained control cells.

Supplementary Figure S4 Proliferation of allogeneic T cells in co-cultures of SC-DCs from HD and MM samples. A representative FACS profile of T cells after 5 days of co-cultures with SC-DCs from both the groups is depicted. Dark green filled histogram represents CD3+ allogeneic T cells only while light green filled histogram represents SC-DCs –CD3+ allo-T cells co-cultures.

Supplementary Figure S5 CCR7 and CCL-19 interaction is essential for DC migration. *In vitro* migration of HD-SC-DCs and MM-SC-DCs toward CCL-19 was significantly reduced only when the mature DCs were treated with anti-CCR7 antibody. Whereas, blocking of other receptors such as CCR1, CCR3, and CCR5 did not show any significant reduction in DCs migration. Data given are mean ± S.E.M *p* ≤ 0.001 (^***^).

Supplementary Figure S6 Sorting of naïve T cells from HD/MM samples. Representative FACS profile and gating strategy for sorting of naïve T cells from apheresis samples of MM patients are shown.

Supplementary Figure S7 Characterization of CTLs from HD-SC-DCs primed against MDA-MB-231-luc-D3H2LN **(A)** Representative FACS profile for the expression of granzyme A, granzyme B, perforin, and CD69 in CTLs generated from HD samples is depicted. **(B)** Levels of IFNγ in the CTLs obtained from three different HD samples were similar.

Supplementary Figure S8 CTLs generated from healthy donor samples showed killing effect against different cancer cell lines *in vitro*. HD-SC-CTLs could be primed against the desired cancer cell lines and had very specific target-killing activity for **(A)** MDA-MB-231-LUC-D3H2LN **(B)** MCF-7 **(C)** A498, and **(D)** HCT-116. The CTLs showed very negligible killing of K562, which was a non-specific target cell line. Data given are mean ± S.E.M.

Supplementary Figure S9 MM-CTLs have reduced expression of serine proteases and activation marker as compared to HD-CTLs: Representative FACS dot plot with isotype control for **(A)** granzyme A **(B)** granzyme B, and **(C)** CD69 in CTLs of both HD samples and MM samples are shown.

Supplementary Figure S10 Analysis of CD8+ T cell subtypes. Naïve effector and memory CD3+CD8+ T cells were analyzed based on marker expression of CD45RA and CD62L. The data shown are for Naïve T cells (CD45RA^+^CD62L^+^) terminally differentiated effector memory cells (CD45RA^+^CD62L^−^) memory cells (CD45RA^−^CD62L^−^) and central memory cells (CD45RA^−^CD62L^+^) from three different HD and MM samples. Only naïve CD8+ T cells were significantly reduced in MM samples. Data given are mean ± S.E.M *p* ≤ 0.01 (^**^).

Supplementary Figure S11 Naïve T cells from MM samples had higher expression of CTLA-4. **(A)** Dot plot showing the representative FACS profiles of naïve T cells from MM and HD samples showing CTLA-4 expression are given. **(B)** Cumulative data from three different samples of HD and MM naïve T cells for CTLA-4 basal expression is given. Data given are mean ± S.E.M *p* ≤ 0.001 (^***^).

Supplementary Figure S12 Expression of costimulatory molecules CD40, CD80, CD83 and CD86 on HD-Mo-DCs, MM-Mo-DCs, HD-SC-DCs and MM-SC-DCs is depicted.

Supplementary Figure S13 MM-Mo-DCs showed significantly lower migration toward CCL-19 as compared to HD-Mo-DCs, HD-SC-DCs and MM-SC-DCs.

Supplementary Figure S14 Percent expression of costimulatory molecules CD40, CD80, CD83 and CD86 on immature and mature HD-Mo-DCs, MM-Mo-DCs, HD-SC-DCs and MM-SC-DCs is given.

## Abbreviations

DCs: Dendritic cells
HSCs: hematopoietic stem cells
Mo: monocytes
HD: healthy Donor
MM: multiple myeloma
Mo-DCs: monocytes derived-DCs
SC-DCs: stem cell-derived DCs
HD-Mo-DCs, healthy donor sample monocyte-derived DCs and MM-Mo-DCs: multiple myeloma sample monocyte-derived DCs
HD-SC-DCs: healthy donor sample stem cell-derived DCs
MM-SC-DCs: multiple myeloma sample stem cell-derived DCs
CTLs: Cytotoxic T cells.
