# Supplementary figures and images for: Autologous Hematopoietic Stem Cells Are a Preferred Source to Generate Dendritic Cells for Immunotherapy in Multiple Myeloma Patients
Source: Front Immunol. 2019 May 21;10:1079. doi: 10.3389/fimmu.2019.01079 (PMC6536579; doi:10.3389/fimmu.2019.01079)

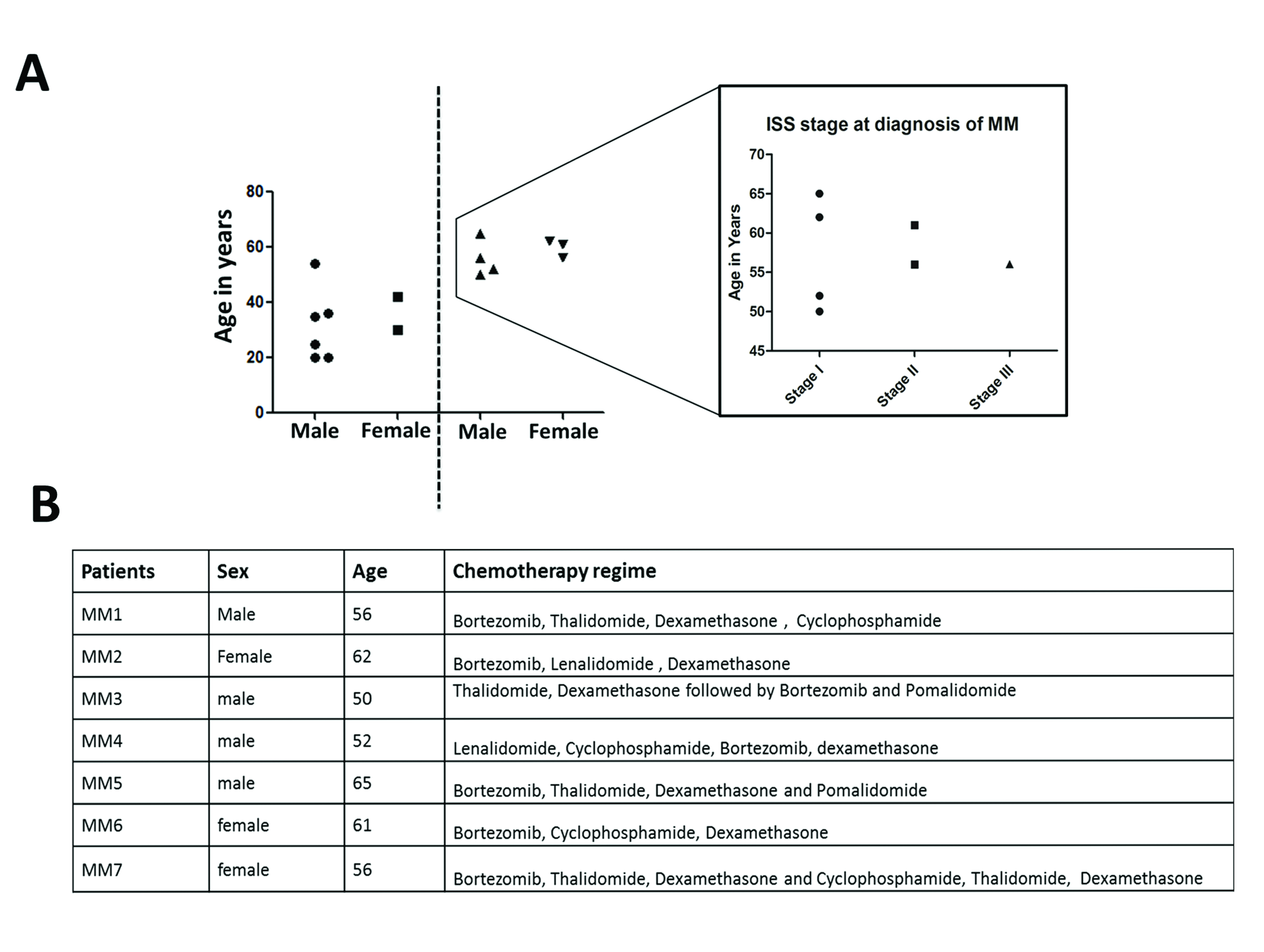

Supplement: Supplementary Figure S1 — Healthy donors and multiple myeloma patients' characteristics. (A) Details of age and sex of the healthy donors, and stage of the disease at the time of the first visit (for MM patients) is depicted for 8 HD and 7 MM samples. (B) chemotherapy regimens of 7 MM samples is tabulated. [file Image_1.TIF]

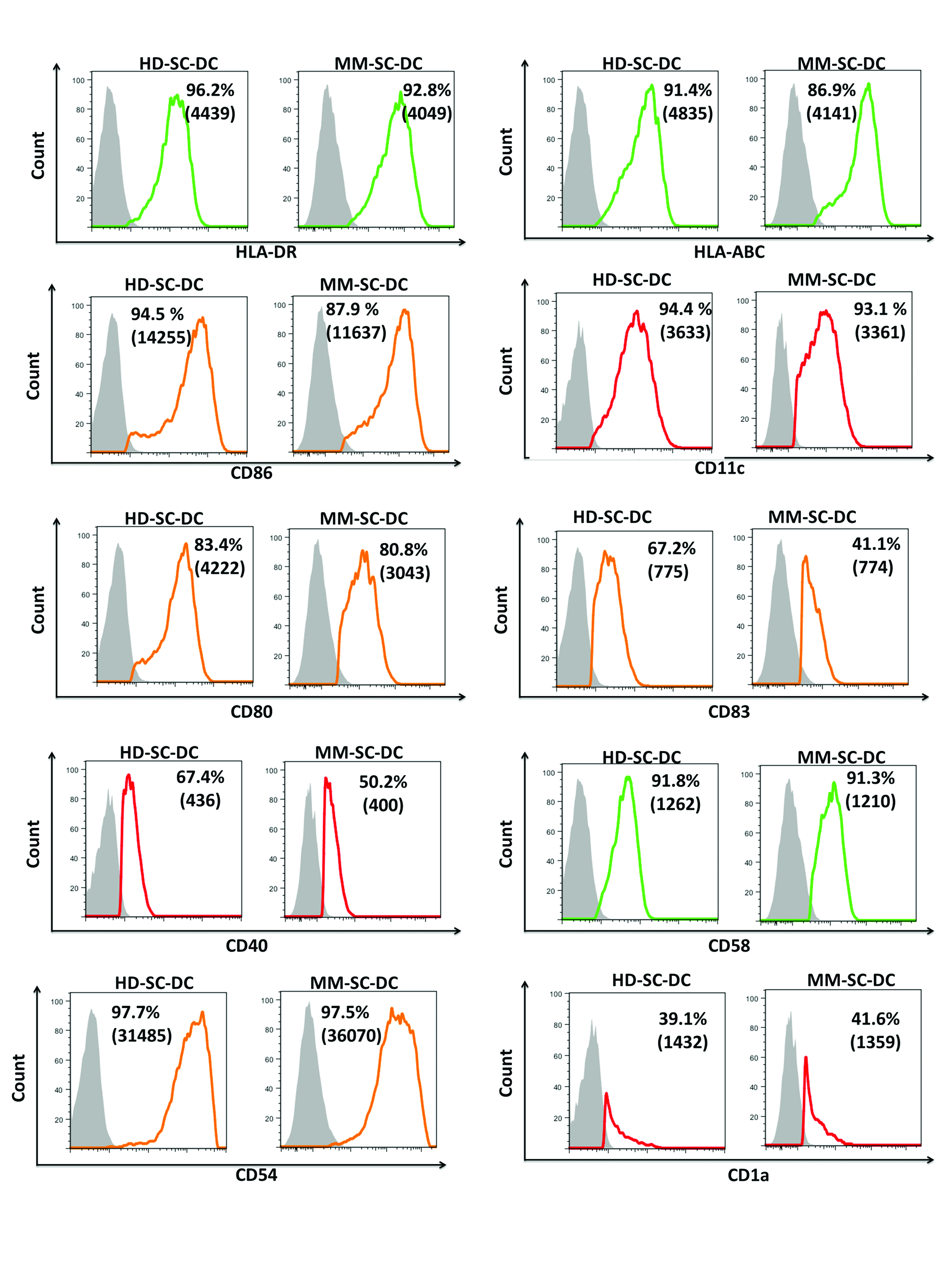

Supplement: Supplementary Figure S2 — Cell surface marker expression on HD-SC-DCs and MM-SC-DCs was comparable. Representatives histogram of DC lineage surface marker (HLA-DR, HLA-ABC, and CD58 in green color; CD86, CD80, CD83, and CD54 in orange color; CD11c, CD40, and CD1a in red color lines) along with their appropriate isotype controls (Gray color filled) are depicted. [file Image_2.TIF]

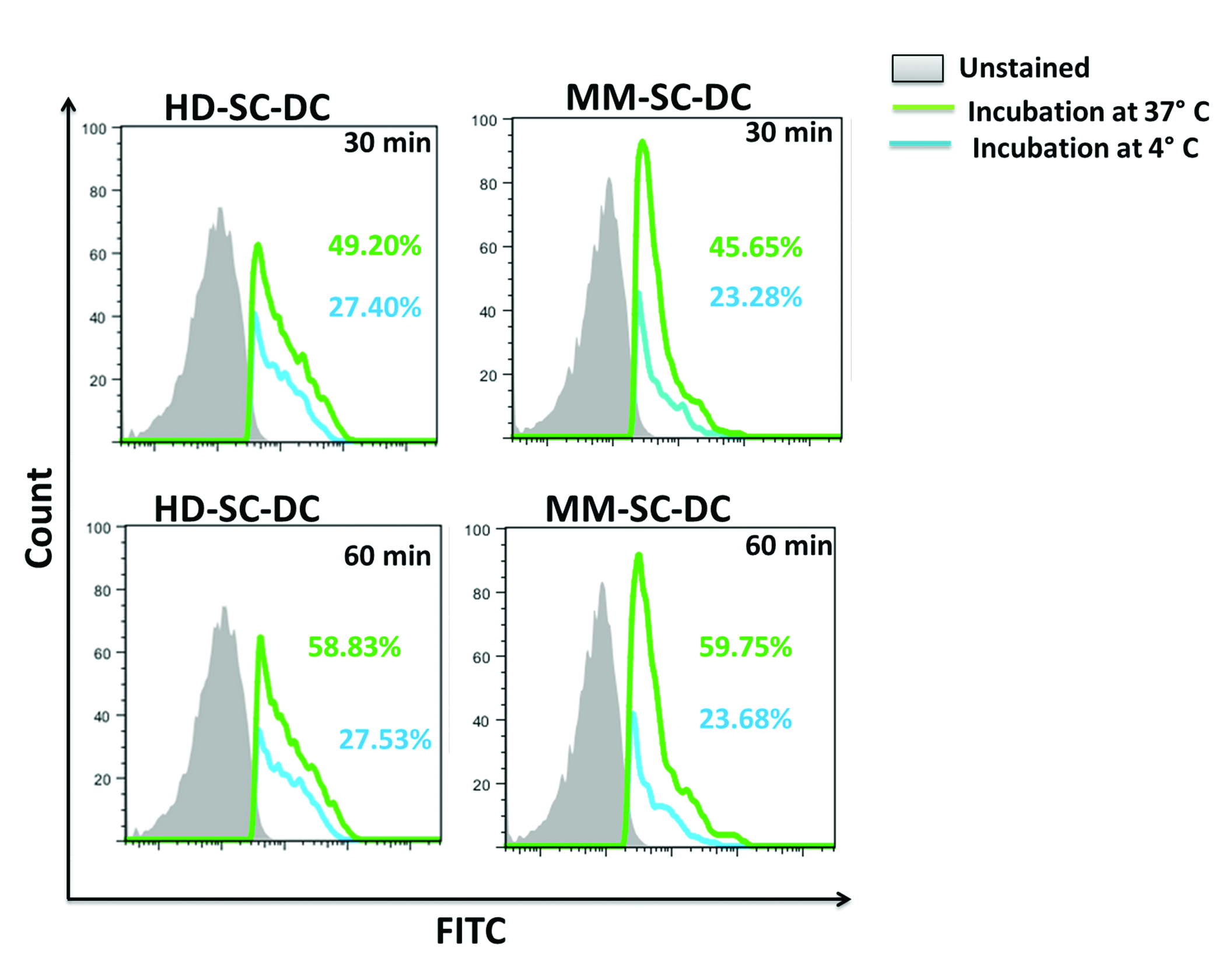

Supplement: Supplementary Figure S3 — MM-SC-DCs and HD-SC-DCs show equivalent dextran-FITC uptake. A representative FACS histogram overlay of dextran- FITC uptake profile from HD-SC-DCs and MM-SC-DCs is depicted. Green line shows uptake at 37°C while the blue line shows uptake at 4°C. Gray shaded histogram shows unstained control cells. [file Image_3.TIF]

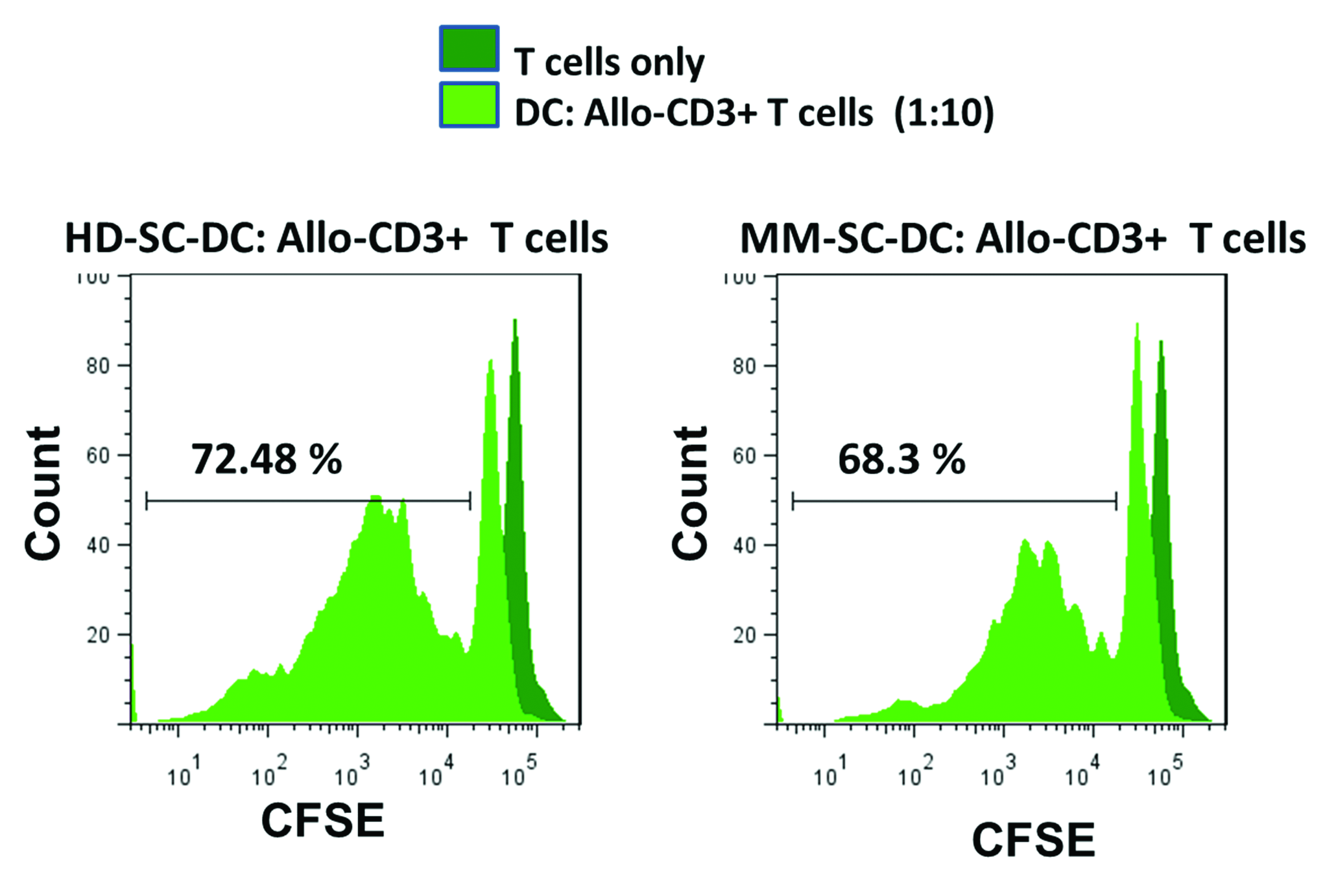

Supplement: Supplementary Figure S4 — Proliferation of allogeneic T cells in co-cultures of SC-DCs from HD and MM samples. A representative FACS profile of T cells after 5 days of co-cultures with SC-DCs from both the groups is depicted. Dark green filled histogram represents CD3+ allogeneic T cells only while light green filled histogram represents SC-DCs –CD3+ allo-T cells co-cultures. [file Image_4.TIF]

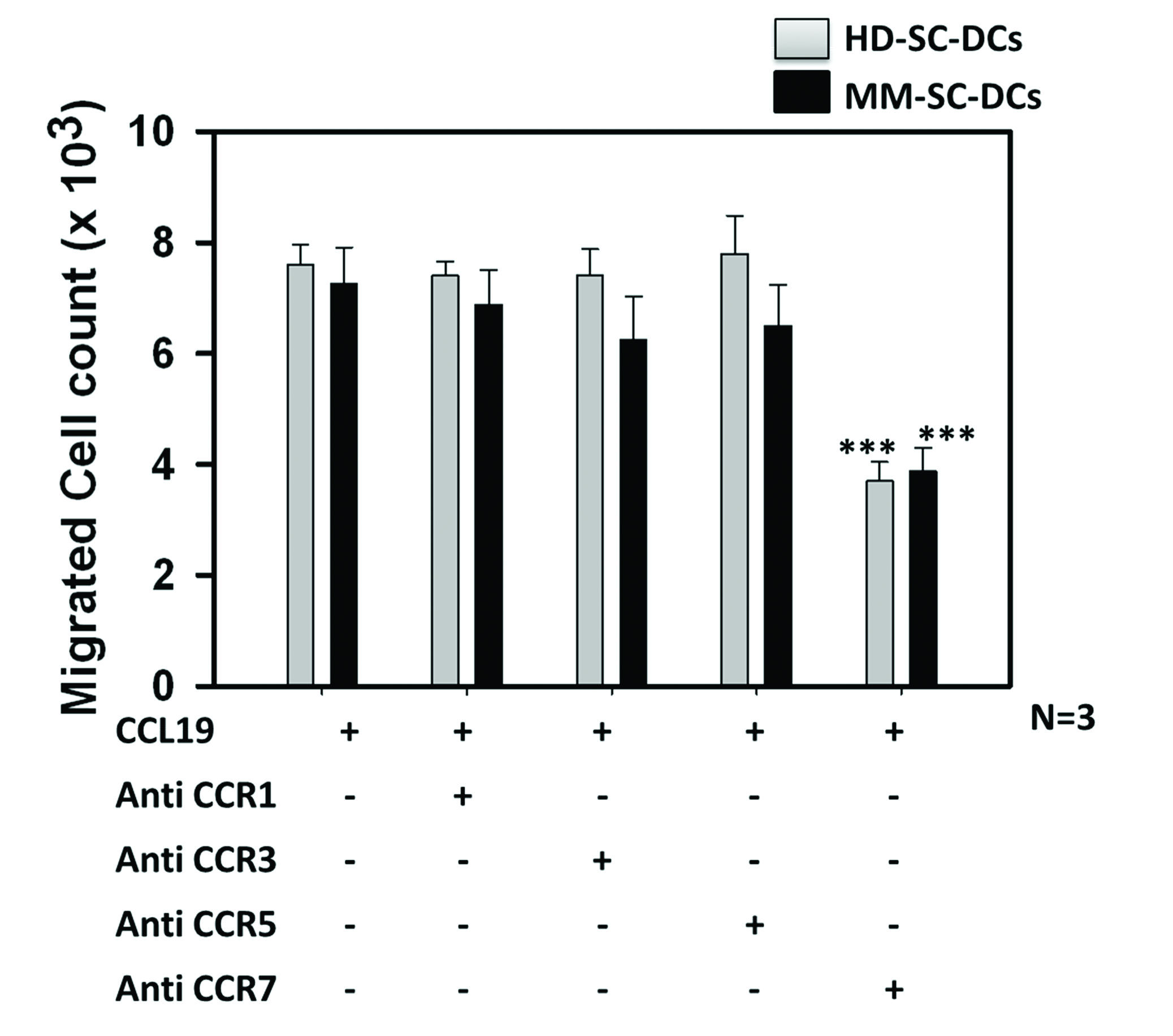

Supplement: Supplementary Figure S5 — CCR7 and CCL-19 interaction is essential for DC migration. In vitro migration of HD-SC-DCs and MM-SC-DCs toward CCL-19 was significantly reduced only when the mature DCs were treated with anti-CCR7 antibody. Whereas, blocking of other receptors such as CCR1, CCR3, and CCR5 did not show any significant reduction in DCs migration. Data given are mean ± S.E.M p ≤ 0.001 (***). [file Image_5.TIF]

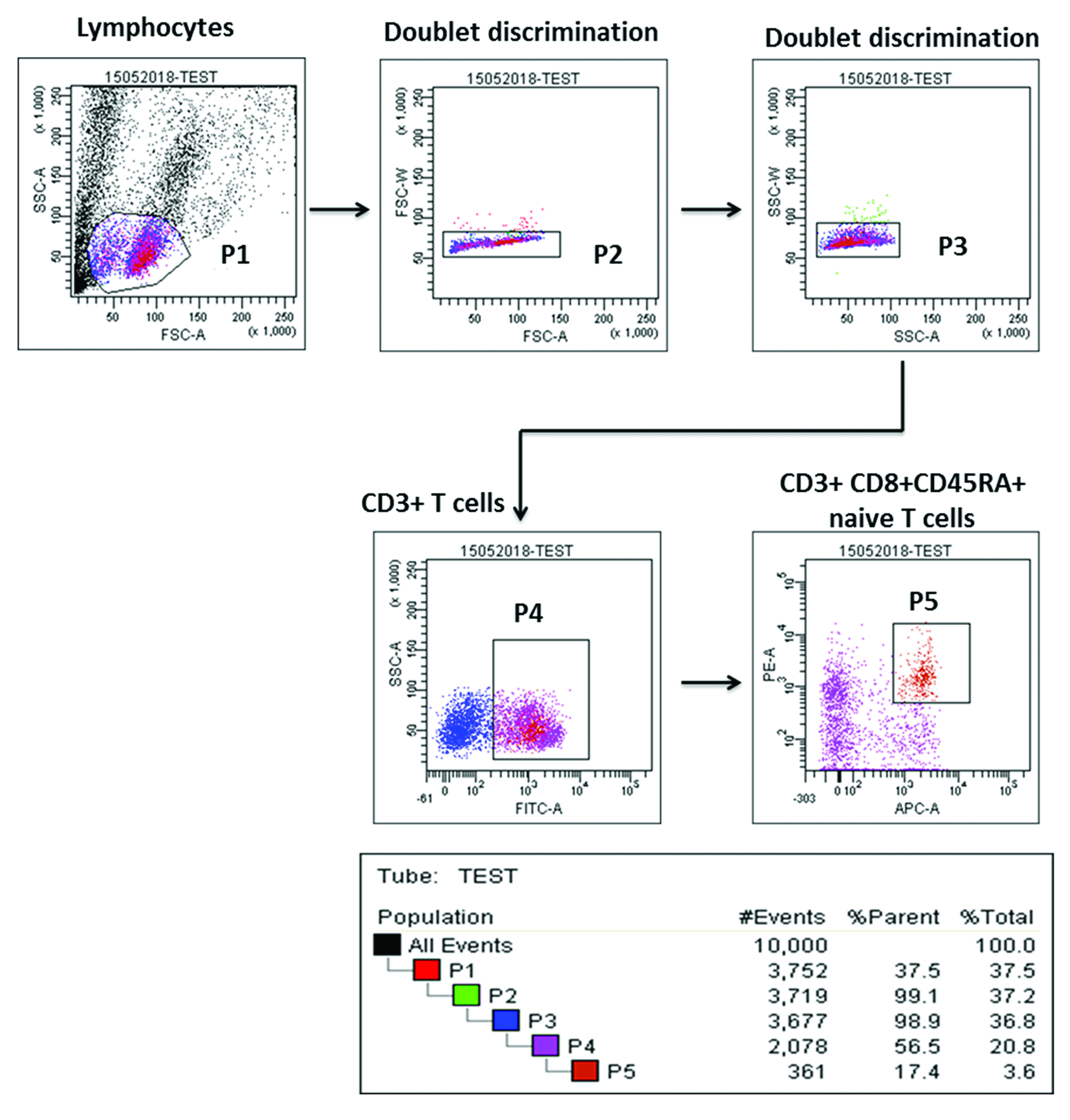

Supplement: Supplementary Figure S6 — Sorting of naïve T cells from HD/MM samples. Representative FACS profile and gating strategy for sorting of naïve T cells from apheresis samples of MM patients are shown. [file Image_6.TIF]

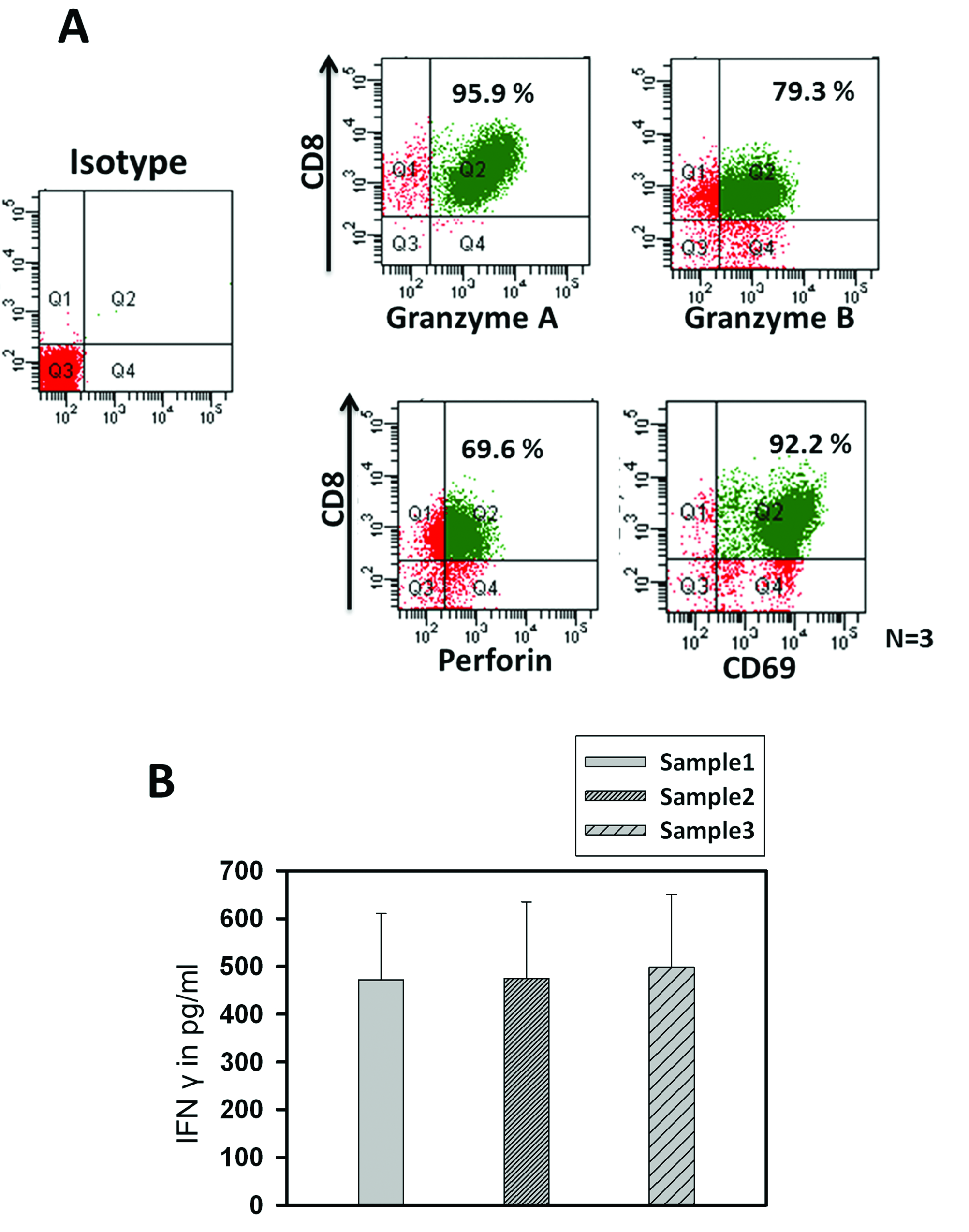

Supplement: Supplementary Figure S7 — Characterization of CTLs from HD-SC-DCs primed against MDA-MB-231-luc-D3H2LN (A) Representative FACS profile for the expression of granzyme A, granzyme B, perforin, and CD69 in CTLs generated from HD samples is depicted. (B) Levels of IFNγ in the CTLs obtained from three different HD samples were similar. [file Image_7.TIF]

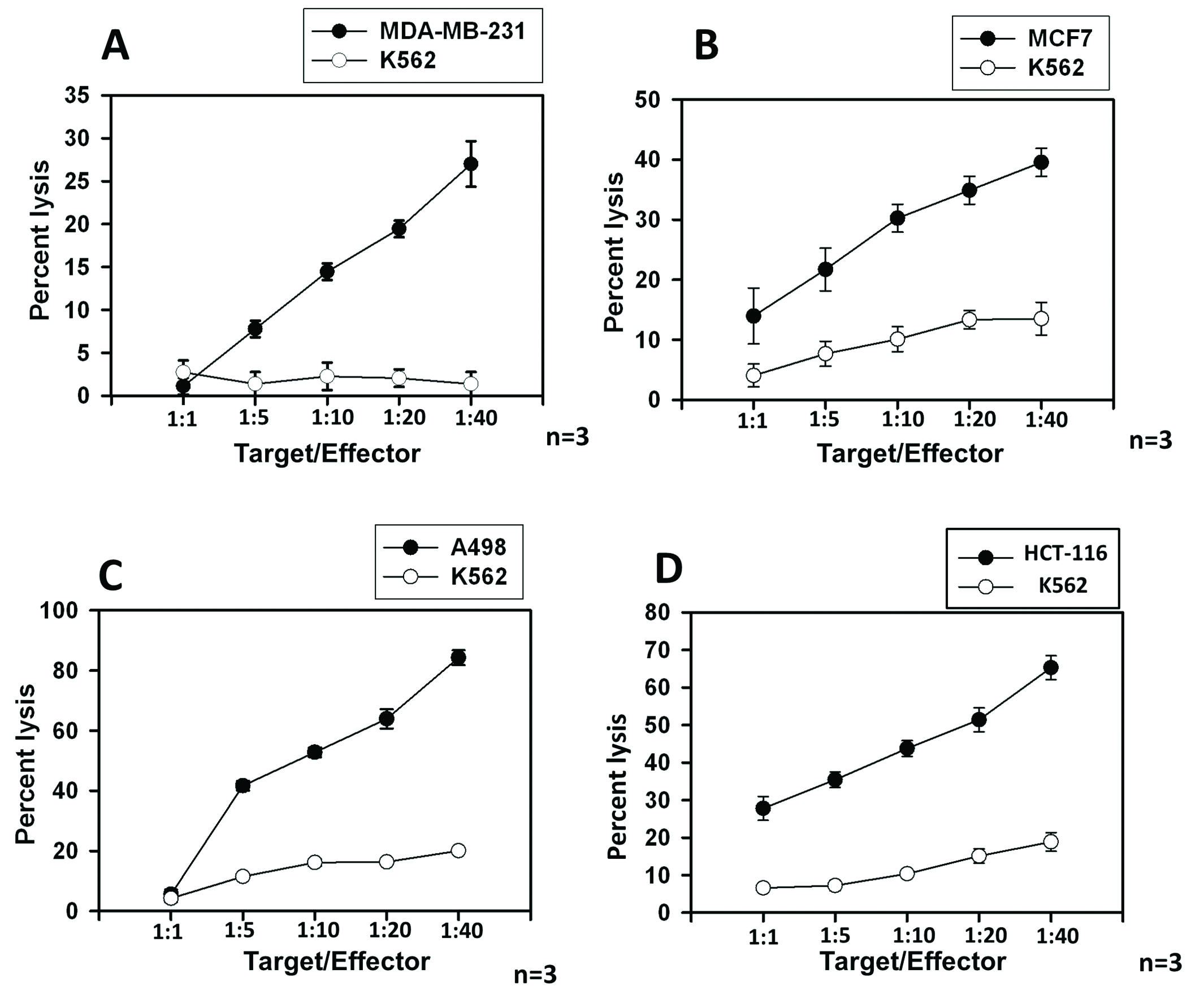

Supplement: Supplementary Figure S8 — CTLs generated from healthy donor samples showed killing effect against different cancer cell lines in vitro. HD-SC-CTLs could be primed against the desired cancer cell lines and had very specific target-killing activity for (A) MDA-MB-231-LUC-D3H2LN (B) MCF-7 (C) A498, and (D) HCT-116. The CTLs showed very negligible killing of K562, which was a non-specific target cell line. Data given are mean ± S.E.M. [file Image_8.TIF]

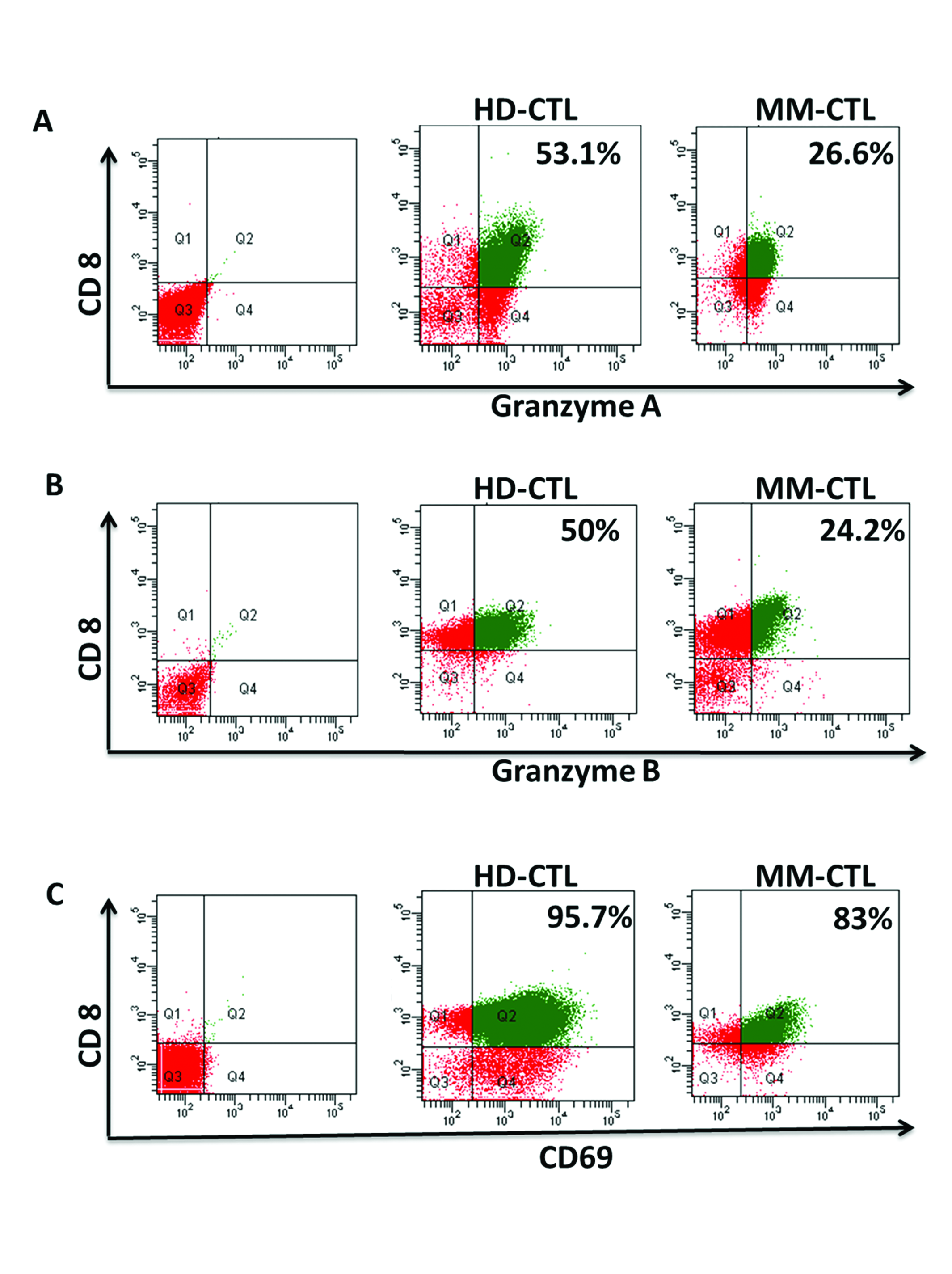

Supplement: Supplementary Figure S9 — MM-CTLs have reduced expression of serine proteases and activation marker as compared to HD-CTLs: Representative FACS dot plot with isotype control for (A) granzyme A (B) granzyme B, and (C) CD69 in CTLs of both HD samples and MM samples are shown. [file Image_9.tif]

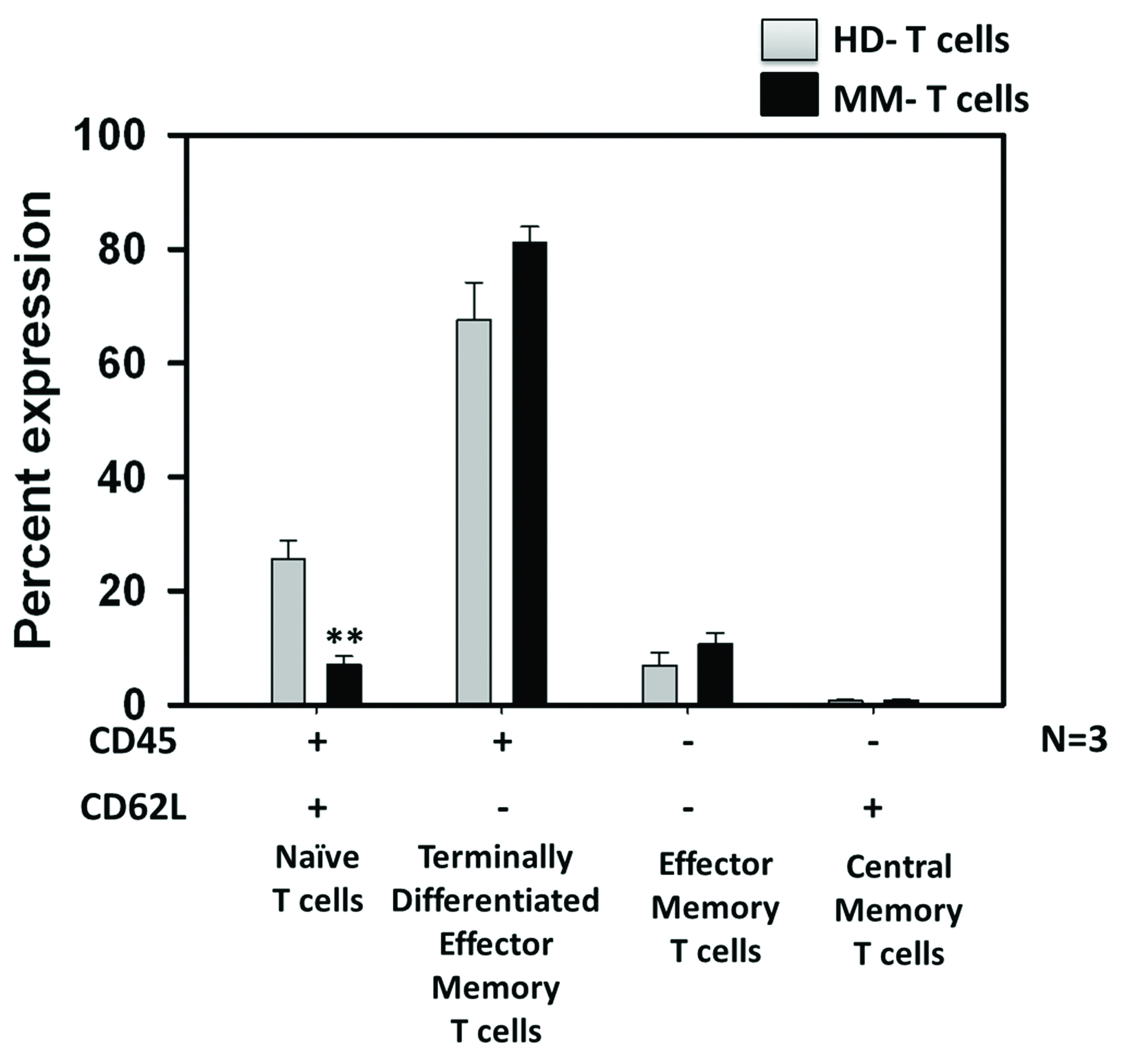

Supplement: Supplementary Figure S10 — Analysis of CD8+ T cell subtypes. Naïve effector and memory CD3+CD8+ T cells were analyzed based on marker expression of CD45RA and CD62L. The data shown are for Naïve T cells (CD45RA+CD62L+) terminally differentiated effector memory cells (CD45RA+CD62L−) memory cells (CD45RA−CD62L−) and central memory cells (CD45RA−CD62L+) from three different HD and MM samples. Only naïve CD8+ T cells were significantly reduced in MM samples. Data given are mean ± S.E.M p ≤ 0.01 (**). [file Image_10.TIF]

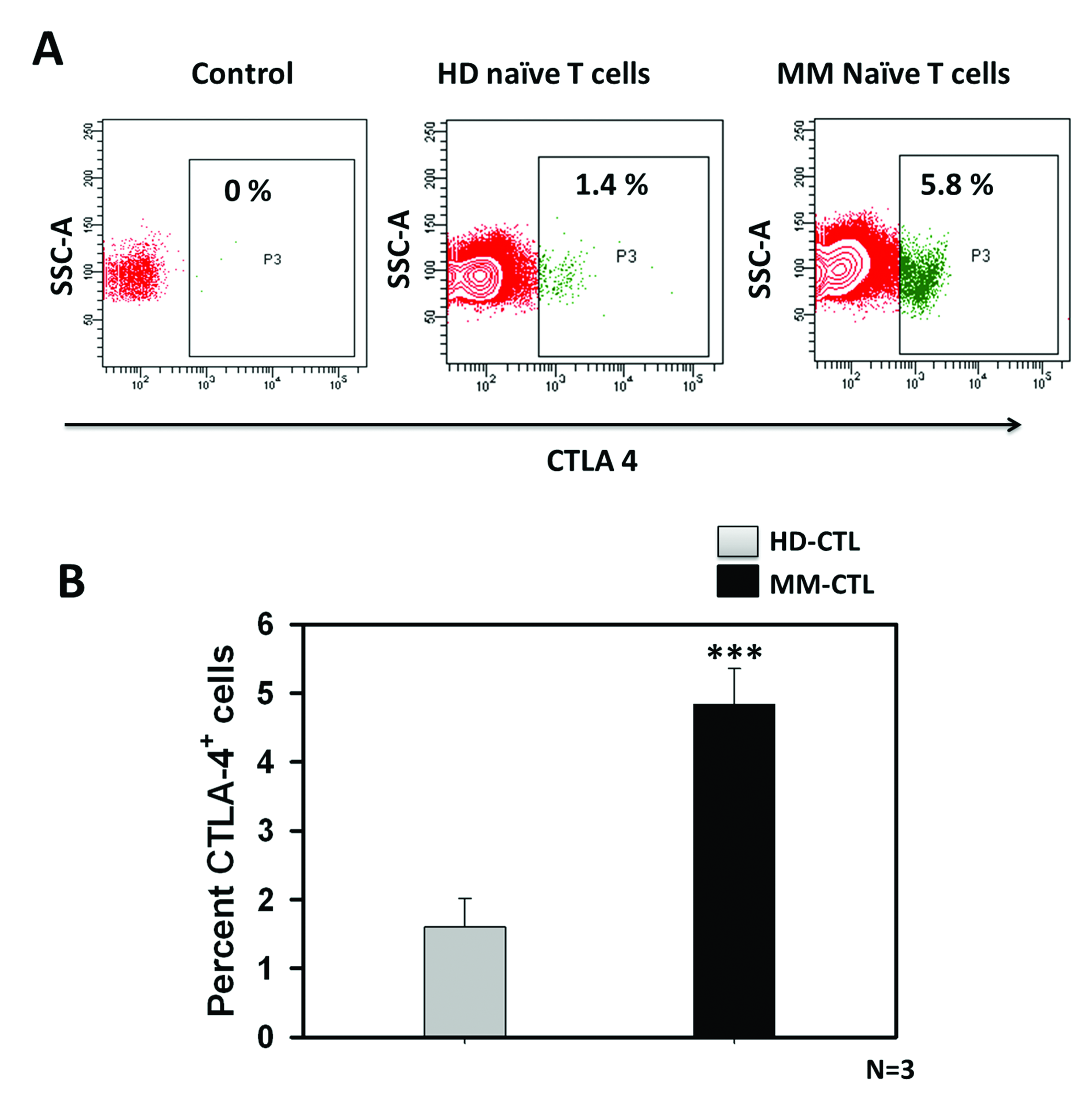

Supplement: Supplementary Figure S11 — Naïve T cells from MM samples had higher expression of CTLA-4. (A) Dot plot showing the representative FACS profiles of naïve T cells from MM and HD samples showing CTLA-4 expression are given. (B) Cumulative data from three different samples of HD and MM naïve T cells for CTLA-4 basal expression is given. Data given are mean ± S.E.M p ≤ 0.001 (***). [file Image_11.TIF]

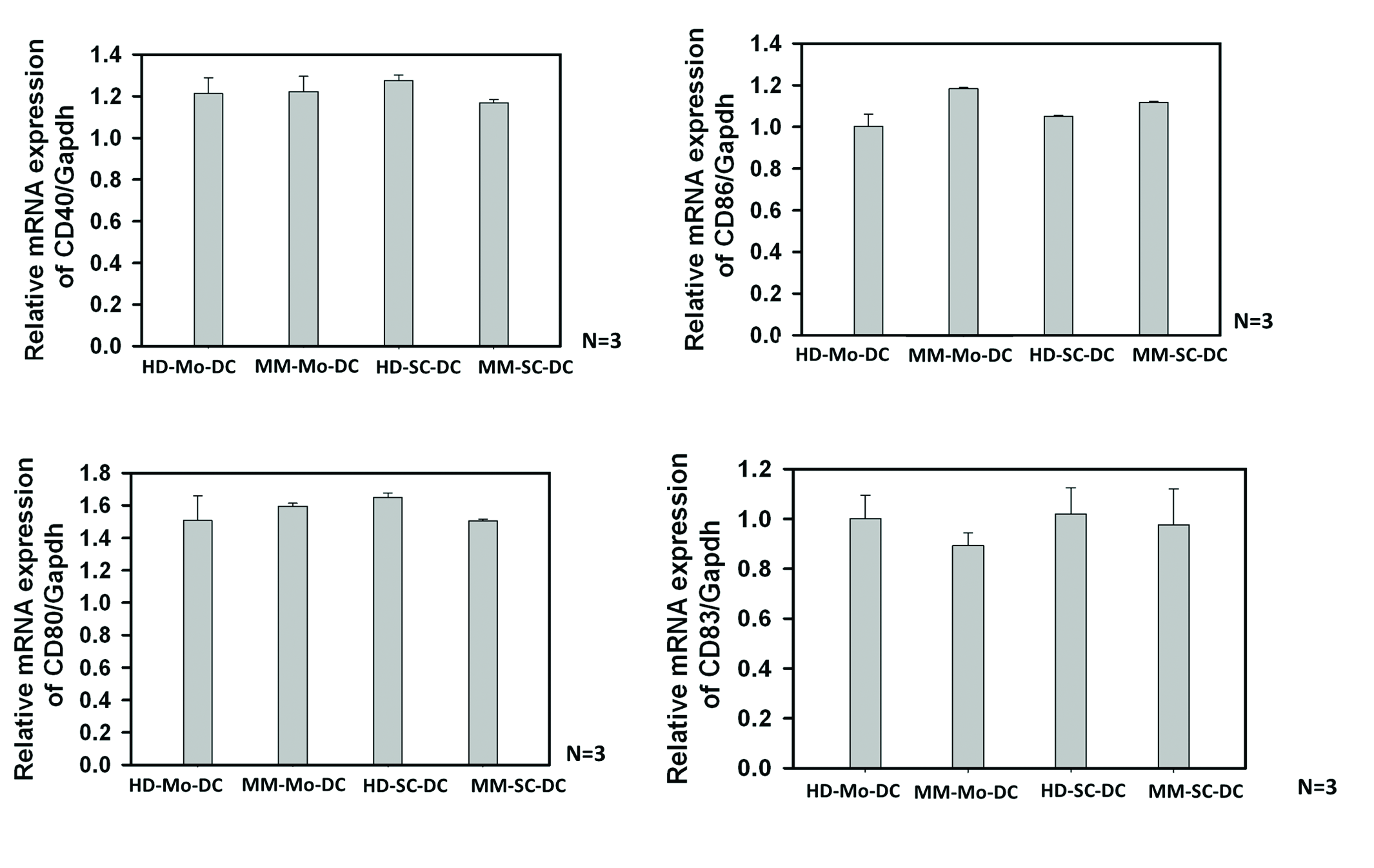

Supplement: Supplementary Figure S12 — Expression of costimulatory molecules CD40, CD80, CD83 and CD86 on HD-Mo-DCs, MM-Mo-DCs, HD-SC-DCs and MM-SC-DCs is depicted. [file Image_12.TIF]

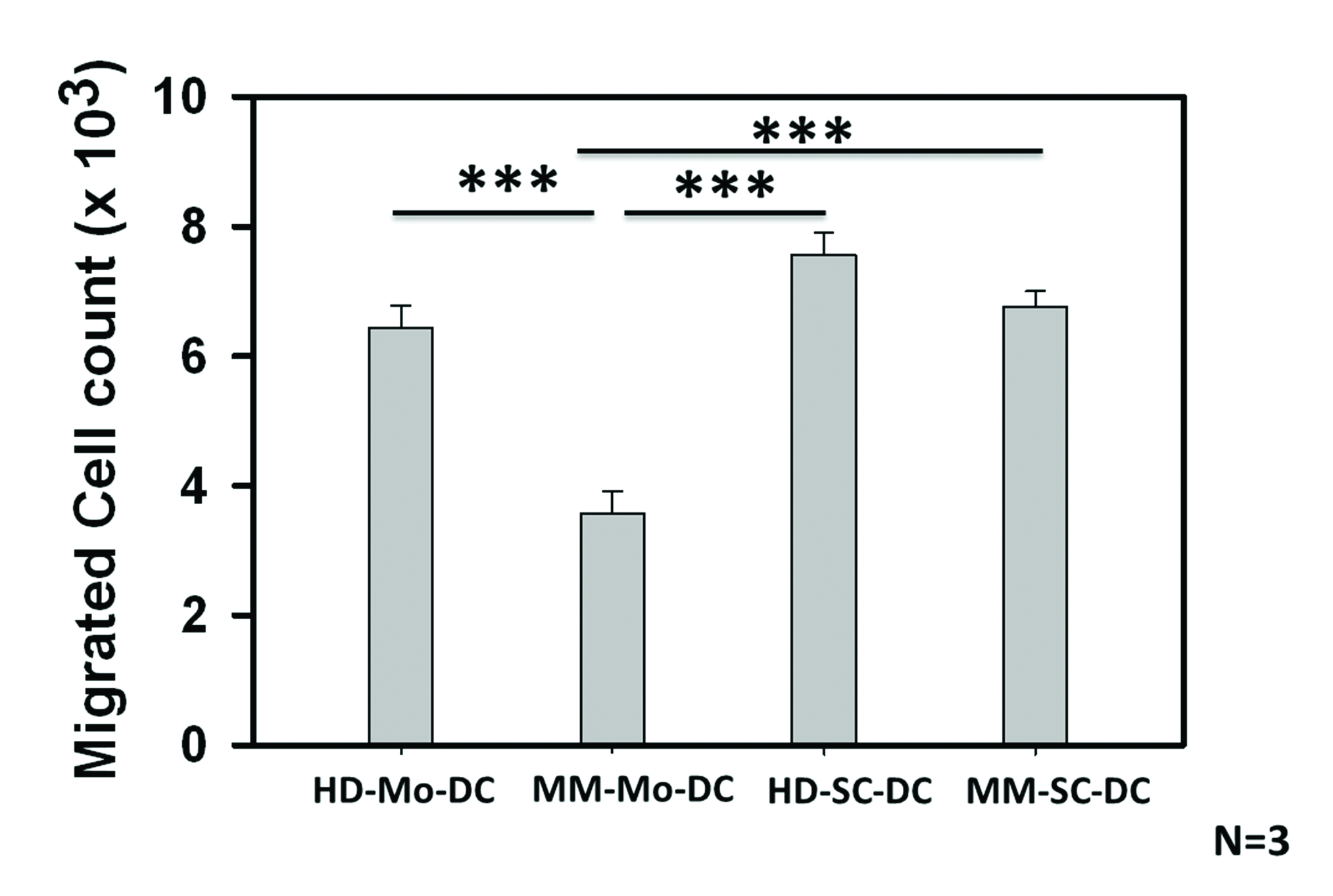

Supplement: Supplementary Figure S13 — MM-Mo-DCs showed significantly lower migration toward CCL-19 as compared to HD-Mo-DCs, HD-SC-DCs and MM-SC-DCs. [file Image_13.TIF]

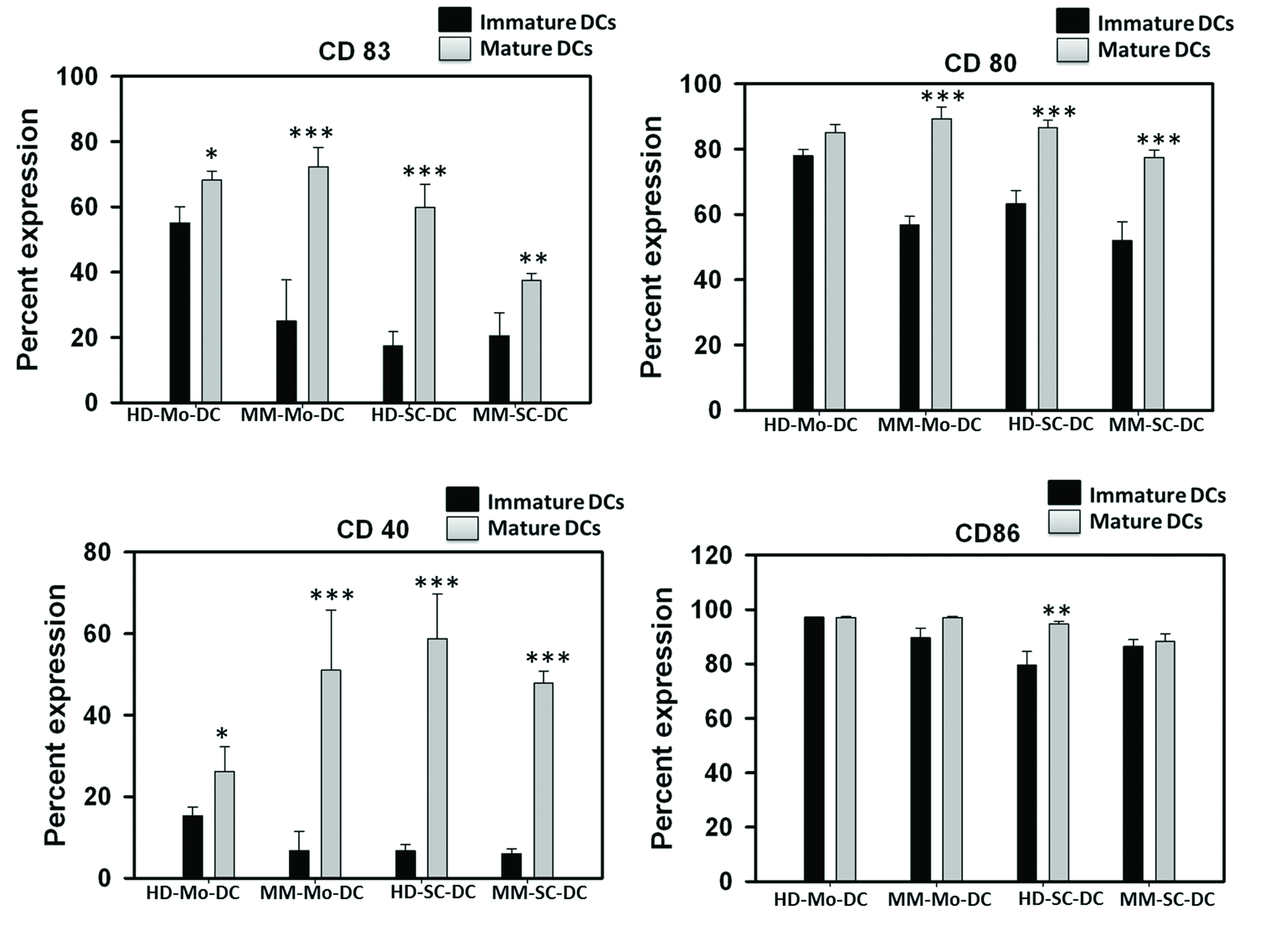

Supplement: Supplementary Figure S14 — Percent expression of costimulatory molecules CD40, CD80, CD83 and CD86 on immature and mature HD-Mo-DCs, MM-Mo-DCs, HD-SC-DCs and MM-SC-DCs is given. [file Image_14.TIF]
